# Effect of health educational intervention on early detection of sickle cell disease among adolescent population in tribal areas of East Singhbhum district, Jharkhand: A study protocol

**DOI:** 10.1371/journal.pone.0345849

**Published:** 2026-04-01

**Authors:** Sarbasree Bhattacharjee, Jarina Begum, Ranjitha S. Shetty, Syed Irfan Ali, Ravi Ranjhan Jha, Anit Kujur, Mohd Amir Ansari

**Affiliations:** 1 Department of Community Medicine, Manipal Tata Medical College, Manipal Academy of Higher Education, Manipal, India; 2 Department of Community Medicine, Kasturba Medical College, Manipal Academy of Higher Education, Manipal, India; 3 Department of Community Medicine, Shaheed Nirmal Mahto Medical College, Dhanbad, Jharkhand, India; 4 Department of Community Medicine, Rajendra Institute of Medical Sciences, Ranchi, Jharkhand, India; Mulago National Referral Hospital, UGANDA

## Abstract

Sickle Cell Disease (SCD) remains a public health challenge in India, particularly among tribal populations. Gaps persist in early detection, awareness, and culturally appropriate interventions, especially among adolescents, a key target group for long-term prevention. The study aims to evaluate the effect of a school-based educational intervention on the awareness, attitude, and early detection of SCD among adolescents in tribal and non-tribal areas of East Singhbhum district, Jharkhand. A sequential explanatory mixed-methods design will be used. In the quantitative phase, adolescents aged 13–19 years from selected government schools will undergo baseline and follow-up assessments of knowledge and attitudes using structured questionnaires. A health education session will be delivered, followed by screening for anemia and SCD using hemoglobin estimation and the solubility test. In the qualitative phase, in-depth interviews with stakeholders, including healthcare providers, ASHAs, school staff, caregivers, and affected adolescents, will explore perceptions, barriers, and feedback related to the SCD and National Sickle Cell Anemia Elimination Mission, 2023 implementation. A quasi-experimental one-group pretest-posttest was conducted among 41 adolescents (13–15 years) from a government school. Knowledge scores increased from 5.31 ± 2.87 to 12.57 ± 2.09 (mean difference: 7.26, 95% CI: 6.10–8.42, p < 0.001). Attitude scores from 1.44 ± 9.21 to 55.83 ± 8.42 (mean difference: 54.39, 95% CI: 50.89–57.89, p < 0.001). Willingness to undergo SCD testing rose from 37% to 83% post-intervention. Stakeholder feedback indicated high acceptability and enthusiasm for similar sessions. The study will provide critical insights into the effectiveness of adolescent-focused health education in enhancing SCD awareness and screening uptake. It will also highlight disparities between tribal and non- tribal populations and inform policy-level recommendations for targeted, equitable interventions. This research will address gaps in adolescents’ SCD awareness and early detection, contributing to the national goal of SCD elimination through evidence-based, community-embedded strategies.

**Clinical Trial Registry of India:** CTRI/2024/12/078542

## Introduction

Sickle Cell Disease (SCD) is a genetic condition affecting the blood, where a mutation causes hemoglobin abnormalities, resulting in red blood cells becoming crescent-shaped. This illness affects millions around the world, particularly among populations of African, Mediterranean, Middle Eastern, and South Asian descent. As per the 2021 Global Burden of Disease report, India ranks among the countries with the greatest impact from SCD-related disability. [1] Globally, more than 300,000 infants are born with SCD every year. [2] The sickle cell gene is commonly found in various tribal communities across India, with prevalence rates ranging from 1% to 40%. [3] In a retrospective study conducted at the Department of Pathology, RIMS, Ranchi, researchers examined cases of hereditary hemoglobin disorders from July 2018 to February 2020. The study involved individuals who tested positive for these disorders using solubility tests and the Naked Eye Single Tube Red Cell Osmotic Fragility Test (NESTROFT), followed by High-Performance Liquid Chromatography (HPLC BIO-RAD variant-II SYSTEM). Out of 2023 cases that were positive in screening, 341 (16.85%) showed no evidence of hereditary hemoglobin disorders in the HPLC analysis. Among the confirmed cases, 1294 (64%) were diagnosed with sickle cell disorder. The study also found the prevalence of other hereditary hemoglobin disorders: Beta Thalassemia trait was present in 301 (15%) cases, Beta Thalassemia major in 63 (3%) cases, HbF in 16 (0.8%) cases, HbE trait in 3 (0.14%) cases, HbD trait and HbE Beta Thalassemia each in 2 (0.09%) cases, and HbE disease in 1 (0.05%) case. [4] It arises due to a genetic alteration in the beta-globin (HBB), which plays a central role in hemoglobin synthesis. This mutation results in the production of hemoglobin S(HbS), which differs from the standard adult hemoglobin (HbA) in structure and function. The presence of HbS causes red blood cells to become inflexible and adhere to each other, taking on a crescent or sickle shape. These distorted cells have difficulty navigating through small blood vessels, disrupting blood flow and contributing to the wide range of complications seen in SCD. [5] The clinical presentation of SCD varies widely, with symptoms ranging from mild to severe. The condition encompasses various forms, including Sickle Cell Anemia (SCA), hemoglobin SC disease (HbSC), and hemoglobin S-beta-thalassemia (which can be further classified into beta-positive or beta-negative types). Rare sub-types also exist. Additionally, individuals with sickle cell trait (HbAS), who carry only one mutated gene, generally remain asymptomatic. Among all types, SCA is the most widespread and is associated with chronic hemolysis, period pain crises, dependence on blood transfusions, and gradual damage to multiple organs. One of the most recognizable features of SCD is the recurrence of vaso-occlusive crises. Painful episodes are caused by the obstruction of blood flow to sickled cells. Patients often deal with chronic fatigue, anemia, and are more susceptible to infections. [6] According to the Census 2001, Scheduled Tribes (STs) constitute 26.3% of Jharkhand’s total population (7,087,068 out of 26,945,829). A significant 91.7% of the ST population resides in rural areas. Gumla district has the highest tribal concentration at 68.4%. Over half of the population in Lohardaga and Paschimi Singhbhum also belongs to tribal communities. Districts like Ranchi and Pakur have ST populations ranging from 41.8% to 44.6%. On the other hand, Kodarma (0.8%) and Chatra (3.8%) report the lowest proportions of ST residents. [7] A study in the Al-Ahsa region of Saudi Arabia assessed Sickle Cell Disease (SCD) awareness among intermediate and high school students, a decade after the introduction of a premarital screening program. The research involved 1,500 students from urban and rural areas, who were surveyed using a questionnaire to gauge their knowledge of SCD. Of the respondents, 79% completed the survey, with a higher proportion of females (62%) compared to males (38%). Most participants were aged 16–19, with a notable number being high school students. Among them, 8.3% had SCD, and 14.3% were unaware of their condition. Overall, 89% of the students had heard of SCD, and those familiar with the disease tended to give more accurate answers. While female students showed slightly better knowledge, this difference was not statistically significant. Age did not significantly impact knowledge levels, although older students generally answered more questions correctly. The study identified several misconceptions about SCD, including its complications and influencing factors. Although most respondents correctly identified common complications like growth failure, jaundice, bone pain, and abdominal pain, there was limited understanding of the effects of extreme weather, strenuous exercise, and tobacco use on SCD. Furthermore, many were unclear about the impact of fluid intake and diet. While 77.7% of students were aware of the premarital screening program and 85.8% recognized its importance, only 25% thought SCD could be cured, and many were uncertain about whether they had been screened. The findings suggest that although general awareness of SCD and its complications is high, there are significant gaps in knowledge regarding preventive measures and the disease’s overall nature. [8] Sickle Cell Disease is associated with high rates of comorbidities and mortality, yet there is limited research on the quality of life (QOL) for adolescents with SCD or Sickle Cell Trait (SCT). A study conducted in Orissa seeks to address this gap by examining QOL among adolescents in the Koraput district of Odisha, an area with a high prevalence of sickle cell hemoglobinopathies but lacking in extensive population-based research. Using a modified QOL scale and snowball sampling, the study evaluated the physical, psychological, and social well-being of 387 adolescents. Results revealed that those with SCD experienced significantly more hospitalizations and lower QOL scores in all areas compared to adolescents with SCT and healthy peers. Although adolescents with SCT are often asymptomatic, they still face challenges that impact their QOL, such as frequent colds and perceived economic pressures. [9] A study conducted in Kampala City, Uganda, employed an analytical cross-sectional approach to assess the prevalence of sickle cell trait (SCT) and factors affecting screening uptake among secondary school students. Data were gathered through semi-structured questionnaires to evaluate students’ knowledge, attitudes, and factors influencing SCT screening. Blood samples from consenting participants were analysed for SCT, with results processed using SPSS software. The study targeted advanced-level secondary school students in Kampala, involving a total of 399 participants, and all ethical approvals and consents were secured before data collection. The findings revealed that 5.8% of the participants had SCT (HbAS). A significant majority (67%) held a negative attitude towards SCT testing, although 89.7% of those who consented to testing were interested in knowing their sickle cell status. Only 1.5% had previously undergone sickle cell testing, and most participants demonstrated moderate understanding of sickle cell disease (SCD) and its screening process. Despite being aware of SCD’s hereditary nature, many participants had misconceptions, such as the belief that sickle cell carriers frequently experienced medical issues and had shorter lifespans. [10] Concerns about stigma and discomfort during testing also influenced their willingness to participate. The study found significant correlations between SCT testing and factors such as knowledge of a partner’s sickle cell status and religious affiliation. Literature reveals that targeted health education programmes are critically needed to address knowledge gaps, particularly in rural and tribal communities. Given this context, the present study aims to evaluate the effect of a structured health educational intervention on early detection of SCD among adolescent students in tribal areas of East Singhbhum district, Jharkhand. The rationale for this study is underlined by the urgent need to implement context-specific, school-based interventions to enhance early diagnosis and disease management among high-risk adolescent populations.

### Research gaps identified

Despite the alarming burden of SCD among tribal communities in Jharkhand, there is a striking lack of early, school-based interventions aimed at equipping adolescents who represent a crucial window for prevention with the knowledge and tools needed for timely detection and action. Most existing research has focused on adult or premarital screening, overlooking adolescents and failing to assess the long-term effects of health education on behaviour change. Additionally, disparities between Scheduled Tribe and non-Scheduled Tribe adolescents in awareness, access, and health-seeking behaviour remain poorly understood. The psycho-social, cultural, and systemic barriers that deter young individuals from screening which is largely unexplored. This study seeks to bridge these critical gaps by implementing a targeted health education and screening program within schools, while capturing stakeholder insights to guide culturally responsive, equity-driven strategies under the NSCAEM, 2023.

### Aim

To assess the effectiveness of a school-based health educational intervention in increasing awareness and early detection of Sickle Cell Disease (SCD) among adolescents in tribal areas of East Singhbhum district, Jharkhand, and to evaluate stakeholder perspectives on the National Sickle Cell Anemia Elimination Mission (NSCAEM),2023

### Objectives

1. Create awareness among adolescents in Government Schools towards sickle cell disease.
  a. Assess the baseline knowledge and attitudes of participants before the Health Education Awareness session.
  b. Assess the effect of awareness sessions on knowledge and attitudes post-session, & three months after the Health Education Awareness session.
  c. Evaluate the feedback of the participants on the awareness session.
  d. Compare the level of awareness and attitude of Scheduled Tribes with non-Scheduled Tribes
2. Conduct school-based screening and early detection for anemia and sickle cell disease, followed by counselling and referral among adolescents of the Government School.
  a. Compare the socio-demographic factors of ST with non-ST adolescents concerning Sickle Cell Disease.
3. Evaluate the perceptions of all stakeholders regarding the National Sickle Cell Anemia Elimination Mission (NSCAEM),2023.
  a. Perspective of service providers towards the NSCAEM,2023.
  b. Perspective of beneficiaries towards the NSCAEM,2023.

## Methodology

### Study design

This study follows a Sequential Explanatory Mixed-Methods Design, consisting of a quantitative phase followed by a qualitative phase. The quantitative component is not cross-sectional, but rather a quasi-experimental pre-test/ post-test design with repeated measures (baseline, immediate post-intervention, and 3-month follow-up assessments).

This phase also includes school-based screening for anemia and SCD. The qualitative component involves in-depth interviews with key stakeholders to explore perceptions, barriers, and contextual factors influencing SCD awareness and implementation of NSCAEM, 2023. The qualitative findings will be used to explain, complement, and contextualise the quantitative results, ensuring full alignment with the overall mixed-methods design. The detailed study protocol is provided in the Supporting Information (S1 File).

### Ethical approval

This study received approval from the Institutional Ethics Committee of Manipal Tata Medical College (IEC number: MTMC/IEC/2024/89). Written informed consent from parents/guardians and assent from adolescent participants will be obtained before data collection. All procedures will follow the ethical guidelines of the Declaration of Helsinki.

### Study setting

This study will be conducted in government schools located in East Singhbhum district, Jharkhand, India.

### Study population & sampling

**Fig 1:**
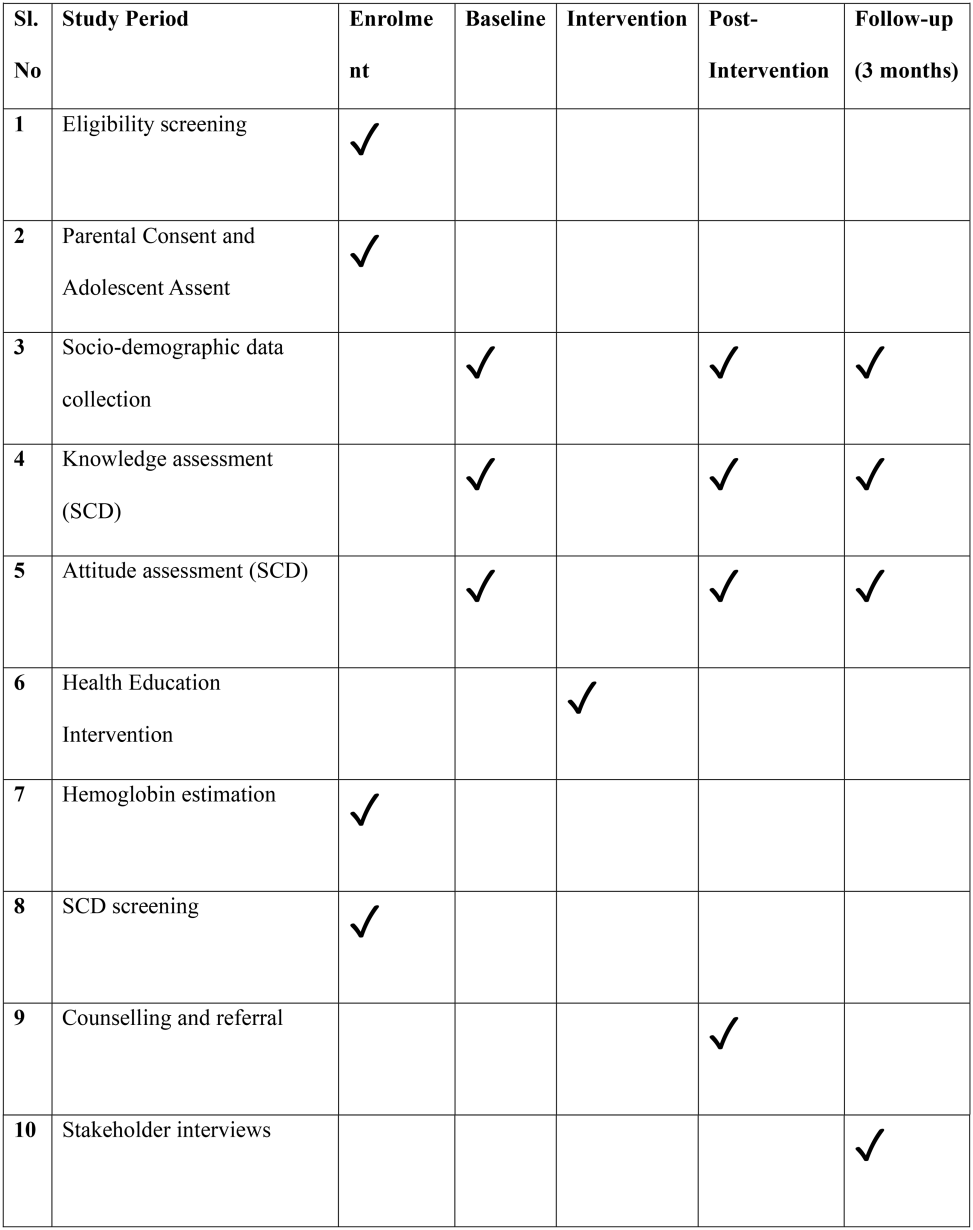
SPIRIT schedule of enrolment, interventions, and assessments.

Adolescents aged between 13–19 years enrolled in Government schools will form the study population. A multi-stage random sampling method will be used, followed by complete enumeration of all eligible students within the selected schools. In the first stage, two blocks in East Singhbhum district will be randomly selected to ensure geographical representation. In the second stage, a complete list of Government schools in each selected block will be chosen using a simple random sampling procedure through the lottery method. Each eligible school will be assigned a unique number, and numbered slips will be drawn without replacement, ensuring transparency and equal probability of selection. Complete enumeration within the selected schools will minimise selection bias and capture the full distribution of knowledge, attitudes, and screening outcomes among adolescents. Schedule illustrating the timing of participant enrolment, health education intervention, screening procedures, and outcome assessments across the study timeline (baseline, post-intervention, and three-month follow-up) is shown in Fig 1.

**Fig 2:**
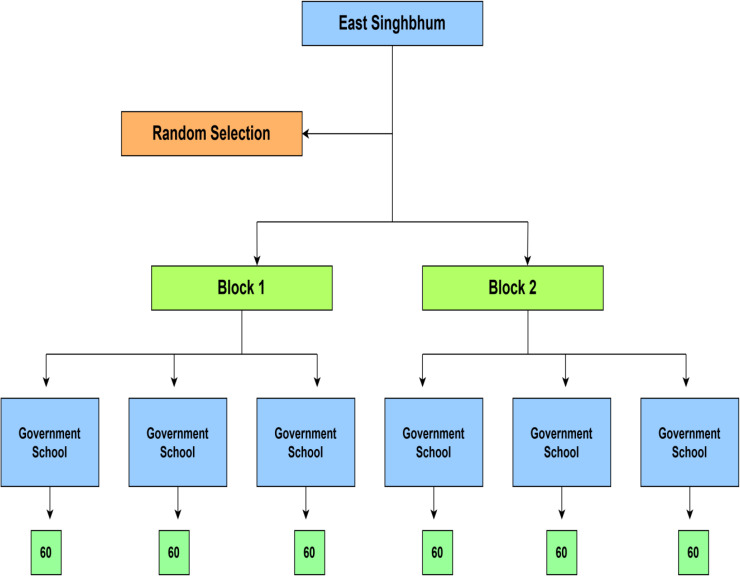
Schematic representation of the school-based sampling strategy across two randomly selected blocks in East Singhbhum district.

This multi-stage random sampling approach will ensure that the final sample is representative, logistically feasible, and appropriate for the school-based mixed methods design, as shown in Fig 2.

**Fig 3:**
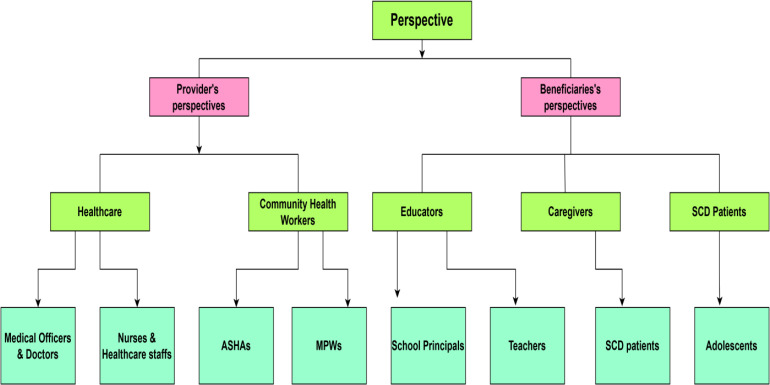
Stakeholder groups whose perspectives will be explored in the qualitative phase of the study.

The qualitative phase will include diverse stakeholder groups, healthcare providers, educators, caregivers, and adolescents with SCD to ensure a comprehensive understanding of contextual perceptions, as shown in Fig 3.

### Intervention

Participants will be receiving a structured health education session focusing on the causes, transmission, symptoms, and prevention of SCD. Each session will involve approximately. 30 participants to ensure an effective interactive session.

### Study status and timeline

Participant recruitment is scheduled to begin in December 2025 and will be completed in phases to align with the sequential explanatory mixed-methods design of the study. The first phase will involve baseline quantitative data collection and delivery of the health education intervention, immediate post-intervention assessments, school-based anemia and SCD screening, and subsequent counselling and referral activities. The final phase will comprise qualitative data collection through in-depth interviews with key stakeholders, conducted after completion of the quantitative phase to ensure integration of findings. The phased data collection process is expected to conclude by August 2027, with data cleaning, analysis, and triangulation of quantitative and qualitative findings planned for August 2026. Final results are anticipated to be available by the end of August 2027. The pilot study results presented in this manuscript were generated solely as part of the preparatory activities for refining the intervention content, tools, and logistics, and are not part of the main study’s dataset.

### Inclusion criteria

Participants will be enrolled from a government school, and only those who provide assent and receive consent from their guardians will be included in the study. Eligible participants should be within the adolescent age range of 13–19 years.

### Exclusion criteria

Participants who plan to transfer to another school before the conclusion of the study or who will be absent during any of the scheduled visits will be excluded from the study.

### Sample size

#### Quantitative phase.

Prevalence/ Screening objective (primary):

To estimate the expected prevalence of sickle-cell trait/ positive screening (assumed p = 10%) with 95% confidence and an absolute margin of error of ±5%, we used the standard single-proportion formula:


$$\text{n}={\text{Z}}^{\text{2}}\cdot \text{p}\cdot (\text{1}-\text{p})/{\text{d}}^{\text{2}}$$

With Z = 1.96, p = 0.10, and d = 0.05, this gives n ≈ 139. This sample provides a reasonably precise estimate of overall trait prevalence in the study population.

Adjustment for cluster sampling (design effect):

Schools were sampled using a multi-stage cluster approach, and students will be enrolled by complete enumeration within selected classes. To allow for clustering (participants within the same school are more similar than between schools), a conservative design effect (DEFF) of 2.0 was applied. This choice is consistent with typical school-based surveys where cluster sizes are moderate and intracluster correlation coefficients (ICCs) for knowledge/ attitude outcomes often range from ~0.01–0.05. (For example, with average cluster size m ≈ 30 and ICC ≈ 0.04: DEFF = 1 + (m − 1)·ICC ≈ 2.16; we therefore used DEFF = 2 as a conservative, pragmatic adjustment.) Applying DEFF raises the required sample to ≈278.

Allowance for loss to follow-up/ non-response:

To accommodate potential attrition and non-response at the immediate post-test or the 3-month retention assessment, we inflated the sample by 20%:

278 × 1.20 ≈ 347.5, rounded to a practical field target of **360 participants** (approximately 12 school sessions × 30 students each, or analogous distribution depending on selected schools)

Power for pre-post knowledge/ attitude changes (intervention objective):

The pilot (n = 41) showed large pre–post improvements in knowledge and attitude. Using conventional sample size rules for paired tests, even a small-to-moderate standardised paired effect size (Cohen’s d = 0.3) requires ~88 participants, and a moderate effect (d = 0.5) requires ~32 participants to achieve 80% power at α = 0.05. With **360 participants**, the study is therefore well powered to detect small-to-moderate within-participant changes in knowledge and attitude following the education session and at 3-month follow-up.

### Justification for the absence of a control group

A classical external control group was not included due to ethical and operational considerations within the Government school system. Withholding a health education intervention related to a high-burden genetic condition from selected students was deemed ethically inappropriate. Additionally, logistical constraints and administrative policies limited the feasibility of randomizing schools to intervention and non- intervention arms.

The study therefore adopts a quasi-experimental, within-participant repeated measures design in which each participant serves as their own comparator across three time points (baseline, immediate post-intervention, and three-month follow-up). While this design does not provide a true external counterfactual, it allows assessment of temporal changes plausibly attributable to the intervention.

We acknowledge that before-after designs without a control group may be vulnerable to threats to internal validity, including maturation effects, history effects, and regression to the mean. Accordingly, several methodological and analytical strategies have been incorporated to mitigate these risks, as detailed below.

**Bias mitigation and counterfactual considerations**

To address potential bias arising from the absence of a classical control group, multiple design-based and analytical safeguards have been incorporated:

- **Baseline measurement prior to intervention:** All primary outcomes are measured before intervention delivery, ensuring temporal ordering and enabling within-individual change estimation.
- **Short-term and sustained follow-up:** Immediate post-intervention and three-month follow-up assessments allow differentiation between transient testing effects and sustained knowledge retention.
- **Adjustment for measured confounders:** Multivariable mixed-effects regression models will be employed to estimate the change in knowledge and attitude scores over time while adjusting for age, sex, tribal status, and baseline knowledge level, socio-economic indicators, and prior awareness of SCD. This reduces confounding due to observable participant characteristics.
- **Cluster level adjustment:** Because students are nested within schools, cluster-adjusted standard errors or random intercept mixed models will be used to account for intra-school correlation.
- **Sensitivity analyses:** Sensitivity analyses will be conducted to assess the robustness of findings to: Exclusion of extreme baseline scores (to reduce regression to the mean effects). Differential attrition at follow-up. Alternative model specifications (parametric vs non-parametric approaches).
- **Assessment of secular trends:** The intervention is delivered over a short and structured time frame within each school, reducing the likelihood of major external secular influences. No concurrent SCD awareness campaigns are planned during the study period; this will be documented.
- **Conservative interpretation of effect estimates:** Findings will be interpreted as evidence of association and temporal change rather than definitive causal proof. Casual language will be avoided, and conclusions will explicitly acknowledge design limitations.

Although a cluster randomized design would offer stronger counterfactual validity, the above strategies enhance internal validity and reduce bias within the ethical and operational constraints of the study context.

#### Qualitative phase.

The study will involve a diverse group of stakeholders, including healthcare providers such as medical officers, doctors, nurses, healthcare staff, community health workers, Accredited Social Health Activists (ASHAs), and Multi-Purpose Workers (MPWs). Educators, including school principals and teachers, as well as caregivers of individuals with sickle cell disease (SCD), including family members, will also be included. Additionally, adolescent research participants diagnosed with SCD will be engaged. Data collection will continue until thematic saturation is reached, that is, until interviews from each stakeholder group no longer yield new themes or insights.

### Data collection methods

The study will use a structured and externally validated questionnaire to assess knowledge and attitudes towards Sickle Cell Disease (SCD), with established reliability demonstrated by Cronbach’s alpha values of 0.722 for the knowledge section and 0.990 for the attitude section, indicating strong internal consistency. The knowledge section will comprise multiple-choice and true/false questions covering the etiology, inheritance pattern, signs and symptoms, preventive measures, and available treatment options for SCD; each correct response will be awarded one point, while incorrect or “don’t know” responses will receive zero points, and total scores will be calculated by summing all correct responses, with higher scores indicating better knowledge. The attitude section will include statements rated on a 5-point Likert scale (strongly agree to strongly disagree) assessing perceptions, stigma, willingness for testing, and perceived importance of prevention; positive items will be scored from 5 to 1, while negatively framed items will be reverse scored, and composite attitude scores will be calculated, with higher scores reflecting more favourable attitudes towards SCD awareness, screening, and prevention. The primary outcome variables will be the knowledge and attitude scores, while independent variables will include socio-demographic characteristics such as age, gender, class/grade, caste/tribal status, parental education, family income, and socio-economic status; covariates will include hemoglobin level categories, prior awareness of SCD, family history of SCD or trait, and exposure to the health education intervention. Participants will also be asked about their preferences for testing locations and their willingness to undergo SCD testing before and after the intervention. These scoring methods will facilitate both within-group (pre- and post-intervention) and between-group (Scheduled Tribe vs. non-Scheduled Tribe) comparisons to evaluate the effectiveness of the health education intervention. This study will employ a multi-phase approach to assess both the biomedical and socio-cultural aspects of SCD among adolescents. It will begin with the administration of a structured questionnaire to all participants, designed to evaluate their knowledge, awareness and attitudes towards SCD. This tool will capture their understanding of the disease’s causes, symptoms and prevention strategies as well as their perceptions and beliefs surrounding SCD. Following this, participants will undergo a two-phase screening process. In the first phase, hemoglobin level assessments will be conducted to evaluate the presence and severity of anemia, which is a critical factor that may affect the diagnosis and clinical presentation of SCD. These assessments will be carried out in designated areas within participating schools, which will be temporarily converted into clean and organized blood collection stations. Using finger prick methods with sterile lancets and capillary tubes, trained lab technicians will collect small blood samples. Hemoglobin levels will be analysed using portable hemoglobin meters, and categorised as normal, mild anemic, moderate anemic and severe anemic. In the second phase, participants will undergo SCD screening through a solubility test conducted in a mobile laboratory set up at the school premises. The test will involve mixing a blood sample with a reagent solution to detect the presence of sickle hemoglobin (HbS), cloudiness in the solution will indicate a positive result. All testing will be conducted under strict hygiene protocols using personal protective equipment (PPE) and proper biomedical waste disposal systems. Participants and/or their guardians will be informed of the results and those testing positive for SCD will receive personalized counselling, health education and referrals to appropriate health facilities for further diagnosis and care. Additionally, in depth interviews will be conducted with various stakeholders including healthcare providers (doctors, nurses, ASHAs, MPWs), educators (principals, teachers), caregivers, and adolescent patients diagnosed with SCD. These interviews will aim to explore personal experiences, barriers to healthcare access, cultural beliefs and perceptions related to SCD diagnosis, prevention and treatment. The qualitative data will be collected until saturation is achieved when no new themes or insights emerge ensuring a well- rounded understanding of the challenges and opportunities for improving SCD care and awareness in the study population.

### Data collection procedure

The study will be conducted in government schools to evaluate the effectiveness of a multi-phase health education and screening intervention for SCD under the framework of the National Sickle Cell Anemia Elimination Mission. Prior to initiation, the research team will obtain ethical clearance from the IEC, permission from the District Education Officer (DEO) and approval from the Welfare officer. School principals will be approached formally and their written consent will be secured following discussions on study logistics and timing.

The study will begin with a knowledge and attitude assessment using structured and validated questionnaire to evaluate students baseline awareness of SCD, its inheritance, symptoms and prevention. Interactive educational sessions will be conducted across participating schools, each engaging around 30 students totalling 360 adolescent participants. These sessions will use culturally tailored IEC materials, visual aids and participatory methods to enhance understanding of SCD. A post-intervention questionnaire will be administered to measure the change in knowledge and attitudes and a three month follow up assessment will evaluate knowledge retention. Participants will then undergo a two-phase screening process. First, hemoglobin level assessments will be conducted using finger prick blood samples and portable hemoglobin meters to detect anemia. This will be followed by SCD screening using the solubility test in a mobile laboratory set up on school premises. Parental consent and participant assent will be obtained in two stages: once for the educational session and baseline questionnaire and again before medical screening procedures. Results will be documented and participants identified with SCD will receive counselling and referrals for further care. To explore community and healthcare system perspectives, in depth interviews will be conducted with stakeholders including healthcare providers (doctors, nurses, ASHAs, MPWs), caregivers, school staff, and adolescent patients. These interviews will explore perceptions, barriers, cultural beliefs, and stakeholder roles in NSCAEM implementation. Interviews will follow a semi-structured format and be recorded, transcribed, and analysed using a phenomenological approach followed by thematic analysis. A comparative analysis across stakeholder groups will also be conducted to identify gaps and divergences in perspectives. Data saturation will guide the final size, expected to include 60 stakeholders. All data, including questionnaire responses, screening results, and qualitative insights will be analysed using statistical and qualitative analysis software. Descriptive and inferential statistics will assess the impact of the intervention and explore correlations between hemoglobin levels and SCD status. Qualitative findings will provide critical insights into real world implementation of NSCAEM revealing challenges and areas for programmatic improvement. The findings will inform future policy and practice aimed at improving adolescent SCD awareness, early detection and care in underserved regions.

### Data management

All data collected during the study will be systematically organized and securely stored to ensure accuracy, confidentiality, and integrity. Quantitative data from questionnaires and screening results will be coded and entered into Microsoft Excel and subsequently analysed. Data entry will be cross-verified by two independent researchers to minimize errors. Each participant will be assigned a unique identification code to anonymize personal information and maintain confidentiality throughout the data lifecycle. For qualitative data, audio recordings of in-depth interviews will be transcribed verbatim and translated into English where required. Transcripts will be anonymized and securely stored for thematic analysis. The study team will adhere strictly to institutional data protection policies and ethical guidelines to ensure the responsible handling, storage, and reporting of all research data.

### Data analysis

Data analysis will be conducted in alignment with the Sequential Explanatory Mixed-Methods Design. Quantitative and qualitative components will be analysed separately and then integrated to generate comprehensive conclusions.

#### Quantitative analysis.

Quantitative data will be analysed using Jamovi version 2.7.5. Because the study follows a quasi-experimental repeated-measures design without a classical control group, analysis will focus on estimating within-participant change over time while adjusting for potential confounding factors. Primary analyses will use linear mixed-effects models with random intercepts for participants and schools to account for repeated measurements and clustering. Fixed effects will include time (baseline, post-intervention, follow-up) and relevant covariates such as age, sex, tribal status, socio-economic indicators, and baseline score values. This modelling framework enables estimation of adjusted change in outcomes over time while reducing bias from observed confounders.

If the model’s assumptions are not met, robust or non-parametric alternatives will be applied. Effect estimates will be reported with 95% confidence intervals.

Comparisons between Scheduled Tribe and non-Scheduled Tribe adolescents will be performed using Chi-square tests for categorical variables and independent t-tests/Mann–Whitney U tests for continuous variables. Cluster effects arising from school-level sampling will be accounted for using cluster-adjusted standard errors. Screening outcomes (anemia levels and SCD status) will be analysed descriptively and cross-tabulated with socio-demographic variables.

All primary analyses are pre-specified in this protocol, while exploratory analyses are explicitly identified and reported as hypothesis-generating.

To further address potential confounding and clustering inherent in the non-randomised school-based design, multivariable modelling approaches will be pre-specified. Knowledge and attitude scores will be analysed using linear mixed-effects models with random intercepts for schools to account for intra-school correlation and repeated measures within participants. Fixed effects will include time and relevant covariates such as age, sex, tribal status, socio-economic indicators, baseline knowledge or attitude levels, and prior exposure to SCD information. Screening uptake will be analysed using mixed-effects logistic regression with adjustment for the same covariates.

Model assumptions, including linearity, normality of residuals, homoscedasticity, and absence of multicollinearity, will be assessed. If assumptions are violated, generalized linear mixed models with appropriate link functions or robust standard errors will be used. In case of convergence issues or model instability, generalized estimating equations with robust sandwich estimators will be employed as an alternative approach. Missing data mechanisms will be evaluated, and if missingness exceeds minimal levels, multiple imputation using chained equations will be applied under a missing at random assumption, with sensitivity analyses conducted to compare results. These procedures are pre-specified to minimise analytical flexibility and strengthen internal validity.

#### Qualitative analysis.

Qualitative data from in-depth interviews will be transcribed verbatim and analysed thematically using Atlas.ti version 23.4. A phenomenological approach will be adopted to explore lived experiences, perceptions, barriers, and contextual influences on SCD awareness and screening. Coding will involve open, axial, and selective coding, and themes will be developed iteratively until saturation is achieved.

#### Mixed-methods integration.

Following a separate analysis of quantitative and qualitative data, findings will be integrated during interpretation using a triangulation approach. Qualitative themes will be used to explain, contextualise, and elaborate on the quantitative results, particularly areas related to acceptability, barriers, cultural beliefs, and stakeholder perspectives. The integrated findings will provide a comprehensive understanding of the effectiveness and feasibility of the school-based health education intervention.

#### Pre- specified outcomes, analysis plan, and exploratory components.

To minimise undisclosed analytical flexibility and ensure transparency, all primary outcomes, and analytical approaches have been pre-specified in this protocol.

Primary quantitative outcomes are changes in adolescent’s knowledge and attitude scores related to Sickle Cell Disease, measured using a structured and validated questionnaire at three time points: baseline (pre-intervention), immediate post-intervention, and three-month follow-up. These outcomes directly address the primary study objective of evaluating the effectiveness of the school-based health education intervention in terms of change in knowledge & attitude.

Other outcomes include change in awareness among the adolescents, willingness to undergo SCD screening, awareness on status of their hemoglobin level & categories, the proportion of participants screening positive for SCD. These outcomes are descriptive and supportive in nature and are not used to draw causal inferences regarding intervention effectiveness.

Other secondary outcomes include the development of IEC materials tailored for providing health education to specific groups; provision of insights into the program’s implementation, effectiveness, and areas for improvement from both service delivery and beneficiary perspectives; a comprehensive understanding of barriers not directly related to the program; and the generation of representative data on adolescents with respect to sickle cell disease (SCD), leading to publication outputs and copyrighting of the developed IEC materials.

To prevent outcome-dependent analysis decisions, the following analytical pipeline has been defined a priori:

- Within-participant changes in knowledge and attitude scores time points will be assessed using paired statistical tests or repeated measures analysis, depending on data distribution.
- Comparisons between Scheduled Tribe and non- Scheduled Tribe adolescents will be conducted using appropriate group comparison tests.
- Cluster adjusted standard errors will be applied to account for school -level screening.
- Effect sizes and 95% confidence intervals will be reported alongside p-values for all primary outcomes.

**Floor and ceiling effects** will be assessed by examining baseline score distributions. If more than 15% participants achieve the minimum or maximum possible scores at baseline, sensitivity analyses will be conducted, and non- parametric methods will be considered where appropriate.

A statistical power analysis was conducted for the intervention component using conservative assumptions. Based on conventional paired- sample calculations, the final sample size of approximately 360 adolescents provides >80% power to detect small-to-moderate standardised changes (Cohen’s d ≥ 0.3) in knowledge and attitude scores at a two-sided α of 0.05, even after accounting for clustering and anticipated attrition.

Certain analyses are explicitly designated as exploratory. These include:

- Identification of socio-demographic predictors of change in knowledge and attitude scores.
- Associations between hemoglobin levels, sickle cell screening outcomes, and baseline awareness.
- Emergent subgroup patterns not specified in the primary objectives.

Exploratory analyses will be clearly labelled as such in all reports and interpreted cautiously, without inferential overreach.

This transparent distinction between confirmatory and exploratory components ensures methodological rigour while allowing appropriate flexibility for hypothesis generation.

#### Insights from pilot study.

Before implementing the full-scale study, a pilot study was conducted to assess the feasibility, cultural appropriateness, and preliminary effectiveness of the proposed school-based health education intervention on SCD. This preparatory step was considered essential for multiple reasons: first, to verify that the intervention could be delivered smoothly within the time constraints and logistical framework of a government school setting; second, to evaluate the clarity, comprehensibility, and cultural sensitivity of the educational content for adolescents from tribal and similar socio-demographic backgrounds; and third, to test the reliability, validity, and operational flow of data collection tools, including the structured knowledge and attitude questionnaire. The pilot study was carried out in Middle School Ghorabandha, East Singhbhum district in Jharkhand, a location chosen for its demographic comparability to the anticipated study sites and its willingness to collaborate. The study employed the same quasi-experimental one-group pretest-posttest design, session content, and teaching aids (including visual materials, videos, and interactive discussions) that were planned for the main research. Activities included obtaining parental consent and participant assent, administering preintervention questionnaires, delivering the structured health education sessions in phased modules, and collecting post-intervention data on knowledge and attitude. Stakeholder feedback was gathered from both students and teachers to identify aspects of the intervention that were most engaging, highlighting any misunderstandings, and capturing suggestions for improvement. As presented in Table 1, the pilot study showed a strong and statistically significant effect of the health education intervention. Knowledge scores increased from 5.31 ± 2.87 at baseline to 12.57 ± 2.09 post-intervention, resulting in a mean difference of 7.26 (95% CI: 6.10–8.42, p < 0.001). Attitude scores improved from 1.44 ± 9.21 to 55.83 ± 8.42, with a mean difference of 54.39 (95% CI: 50.89–57.89, p < 0.001). Willingness to undergo SCD testing increased from 37% (15/41) to 83% (34/41). These effect sizes demonstrate that the intervention had a substantial positive impact on adolescents’ knowledge, attitudes, and intention to participate in SCD screening, supporting its relevance for the full-scale study.

**Table 1:** Summary of key outcomes from the pilot study on the health education intervention for Sickle Cell Disease (SCD).

Outcome measure	Pre-intervention Mean ± SD	Post-intervention Mean ± SD	Change Mean ± SD	p-value
Knowledge	5.31 ± 2.87	12.57 ± 2.09	+7.26	<0.001
Attitude	1.44 ± 9.21	55.83 ± 8.42	+54.39	<0.001
Willingness to undergo SCD testing	37% (15/41)	83% (34/41)	+46	

#### Feedback from stakeholders.

In addition to the quantitative findings, qualitative feedback gathered from both students and teachers offered valuable insights into the perceived effectiveness of the health education intervention. This feedback enriched the overall evaluation by highlighting participants’ engagement, understanding, and willingness to take further action. Illustrated quotes from students and teachers are presented in Table 2, capturing their perspectives.

**Table 2:** Verbatim feedback from research participants and teachers.

Research participants	Teachers
“मुझे पहले सिकल सेल बीमारी के बारे में कुछ नहीं पता था। अब मुझे समझ आ गया कि यह कितनी जरूरी है। यह सत्र बहुत अच्छा था।” “I didn’t know about sickle cell disease before. Now I can understand how important it is. The session was very interesting. ”	“यह बच्चों के लिए बहुत अच्छा सत्र था। वे ध्यान से सुन रहे थे और समझ भी रहे थे।” “This was a very useful session for the children. They were actively participating and understood the topic well.”
“मैडम ने फोटो और वीडियो से समझाया, इसलिए जल्दी समझ आ गया। ऐसे और सत्र होने चाहिए।” “I liked how Ma’am explained everything with pictures and videos. I could understand easily. We can have more sessions like this.”	“स्कूल में हम ऐसे विषयों पर बात नहीं कर पाते। इस सत्र के लिए धन्यवाद।” “We often don’t have time to discuss such health topics in school. Thank you for taking the initiative.”
“मुझे सवाल-जवाब वाला हिस्सा अच्छा लगा। मेरे सवालों के जवाब मिल गए। मज़ा भी आया और सीख भी मिली।” “I liked the question-answer part. I was able to clarify my doubts. It was fun and we learned a lot too.”	“अगर सिकल सेल की जांच की योजना हो, तो हम मदद करेंगे और स्कूल में करवाएँगे।” “If there is any plan to conduct screening for sickle cell, we are more than willing to support it and help organize it in our school.”
“अब मैं अपने माता-पिता और दोस्तों को इस बीमारी के बारे में बताऊँगा। जाँच कराना ज़रूरी है।” “Now I will tell my parents and friends about this disease. It’s important to get tested.”	“बच्चों में इस विषय को लेकर सच में दिलचस्पी थी। अगर हर कुछ महीने में ऐसा सत्र हो, तो बहुत अच्छा रहेगा।” “We saw a genuine interest in the students. Repeating such sessions every few months would be very helpful.”

### Expected outcome

The study is expected to yield evidence of temporal changes in knowledge, attitudes, and screening uptake following the intervention, while acknowledging limitations inherent to non-randomized designs.It will generate baseline data on adolescents’ knowledge and attitudes toward SCD, and the health education intervention is anticipated to enhance their awareness and significantly influence attitudes. The school-based screening will facilitate early detection of anemia and SCD, enabling timely referral and counselling. The study will also identify disparities in awareness and health outcomes between Scheduled Tribe (ST) and non-Scheduled Tribe (non-ST) adolescents. Feedback from participants will inform the development and refinement of culturally and linguistically appropriate information, education, and communication (IEC) materials. Additionally, perspectives gathered from key stakeholders will provide insights into the strengths and limitations of the National Sickle Cell Anemia Elimination Mission (NSCAEM), 2023, and help in identifying barriers to healthcare access, stigma, and misinformation related to SCD in the community. In the long term, the study is expected to contribute to the integration of targeted SCD education into the school curriculum, particularly in tribal-dominated regions like Jharkhand. The early identification and tailored counselling based on screening outcomes may support a reduction in the disease burden over time. The evidence generated will be instrumental in formulating policy recommendations aimed at optimizing the implementation and reach of national programs like NSCAEM,2023. Moreover, the study will help in designing strategic, population-specific interventions to address the unique needs of tribal and underserved communities. The data and educational tools developed through this research will support future scholarly publications and may also contribute to the creation of copyrightable IEC materials for wider dissemination and use in public health initiatives.

### Importance of proposed research investigation and mention the link to relevant SDG

The proposed research will address a significant public health issue by focusing on SCD, a genetic disorder that disproportionately affects tribal communities in India. Despite the high disease burden in states like Jharkhand, awareness about SCD remains low, and access to timely diagnosis and appropriate management is inadequate. The study will aim to bridge that gap by implementing a structured, school-based health education and screening intervention tailored specifically to adolescents aged 13–19 years in Government schools. Through this approach, the study will not only promote the early identification of anemia and SCD but will also empower young individuals with knowledge that may influence future health decisions, including marriage and reproduction. By comparing Scheduled Tribe and non-Scheduled Tribe populations, the study will also identify health inequities and provide a foundation for developing targeted, inclusive health strategies. The study will generate primary data on knowledge levels, attitudes, and screening outcomes in a previously under-researched adolescent population. It will also explore perceptions and implementation challenges of the NSCAEM,2023, through in-depth stakeholder engagement. These insights will be critical for informing program improvements and guiding policy decisions at the local and national levels. Furthermore, the study will contribute to the creation of culturally appropriate Information, Education, and Communication (IEC) materials, which may be adapted and scaled across similar tribal-dominated regions. In terms of its alignment with global goals, this research will directly contribute to several Sustainable Development Goals (SDGs). It will promote SCD3: Good Health and Well-being by facilitating early diagnosis, prevention, education around SCD, and reducing morbidity and mortality. By embedding health education within schools, it will support SGD4: Quality Education, fostering lifelong learning and health literacy among adolescents. The study’s gender-sensitive design will help ensure both girls and boys benefit equally, thereby contributing to SDG 5: Gender Equality. It focuses on underserved tribal populations and will address health disparities and promote SDG 10: Reduced Inequalities by identifying and responding to the unique needs of vulnerable groups. Finally, the research will foster intersectoral collaboration among educational institutions, healthcare systems, tribal welfare departments, and policy bodies, aligning with SDG 17: Partnerships for the Goals. Overall, this research will serve as a model for community-embedded, evidence-based health promotion, with potential for long-term public health impact and policy relevance, particularly in resource-limited tribal regions of India.

### Patient and public involvement

Participants were not involved in the initial design or development of the research questions, outcome measures, or study protocol due to time constraints and the exploratory nature of this study. Nevertheless, post-intervention feedback was systematically obtained from adolescent participants and school teachers, providing valuable insights into the perceived relevance, acceptability, and impact of the intervention. Teachers and school staff, as critical stakeholders in adolescent health education, played an active role during the implementation phase. Their engagement contributed to assessing the feasibility of intervention delivery in real-world school settings and highlighted key considerations for future adaptation and scale-up.

While formal PPIs were not incorporated in this study, its conceptualization was rooted in the recognition of unmet needs and gaps in awareness among adolescents regarding SCD. We plan to include adolescents and community representatives in the co-design of expanded interventions and strategies to improve engagement and sustainability.

## Supporting information

S1 File File Original Study Protocol. (DOCX)
