# Supplementary material for: Effect of health educational intervention on early detection of sickle cell disease among adolescent population in tribal areas of East Singhbhum district, Jharkhand: A study protocol
Source: PLoS One. 2026 Apr 1;21(4):e0345849. doi: 10.1371/journal.pone.0345849 (PMC13042829; doi:10.1371/journal.pone.0345849)

# Effect of Health Educational Intervention on early detection of Sickle Cell Disease among adolescent population in tribal areas of East Singhbhum district, Jharkhand

PhD Proposal Submitted to

**Manipal Academy of Higher Education, Manipal , India**

By

**Sarbasree Bhattacharjee**

**Dr. T.M.A Pai Scholar(PhD) 245500203**

**Community Medicine**

**Manipal Tata Medical College, Jamshedpur**

Under the Supervision of

# Dr. Jarina Begum

**Professor, Department of Community Medicine Manipal Tata Medical College, Jamshedpur, Jharkhand 831017**

Name of the Co-Supervisor

**Dr. Ranjitha S Shetty**

**Additional Professor, Department of Community Medicine & Coordinator, Centre for Indigenous Population**

**Kasturba Medical College , Manipal, MAHE, Manipal, Karnataka**

**Community Medicine Manipal Tata Medical College**

1. **Introduction**

“Sickle cell disease (SCD) is an inherited blood disorder that results in the formation of abnormal hemoglobin, leading red blood cells to adopt a sickle or crescent shape. This condition impacts millions globally, especiatablly individuals of African, Mediterranean, Middle Eastern, and South Asian heritage. According to the Global Burden of Disease Study 2021, India is one of the nations with the highest disability burden associated with sickle cell disease.”(1)

“The root cause of SCD is a genetic mutation in the beta-globin gene (HBB), which is responsible for hemoglobin production. This mutation leads to the creation of an abnormal type of hemoglobin known as hemoglobin S (HbS), differing structurally from normal adult hemoglobin (HbA). HbS makes red blood cells rigid and sticky, causing them to deform and impair their passage through small blood vessels. The altered shape of these red blood cells is linked to the numerous health issues that accompany SCD.”(2) SCD presents with a range of symptoms that can differ in intensity among patients. “Under the umbrella of SCD, multiple subtypes exist, such as sickle cell anemia (SCA), hemoglobin SC disease (HbSC), and hemoglobin sickle-beta-thalassemia, which can be classified as either beta-thalassemia positive or beta-thalassemia negative. In addition to these, there are several other less common forms of SCD. It’is also crucial to highlight the sickle cell trait (HbAS), which involves a heterozygous mutation and typically does not manifest clinical symptoms. Sickle cell anemia is the most prevalent subtype of SCD, marked by chronic hemolytic anemia that often necessitates blood transfusions, triggers pain episodes, and results in progressive organ damage.”(3)

A key feature is the frequent occurrence of intense pain episodes, termed vaso-occlusive crises, caused by the blockage of blood flow in small vessels due to sickled red blood cells.”(4) “Individuals with SCD) often experience tiredness, anemia, and a heightened risk of infections.”(5)

Jharkhand hosts 32 distinct tribal groups, residing across different parts of the state. These tribes were categorized by anthropologist Lalita Prasad Vidyarthi based on their cultural practices. His classification is as follows:

Hunter-Gatherer Tribes: Birhor, Korwa, Hill Kharia Shifting Cultivators: Sauria Paharia

Traditional Artisans: Mahli, Lohra, Karmali, Chik Baraik

Settled Farmers: Santhal, Munda, Oraon, Ho, Bhumij, and others.

“As per the 2001 census, the population of Scheduled Tribes (ST) in Jharkhand is 7,087,068, comprising 26.3% of the state's total population (26,945,829). A large portion of the ST population (91.7%) lives in rural areas. Gumla district has the highest concentration of STs at 68.4%, while more than 50% of the population in Lohardaga and Pashchimi Singhbhum districts belong to ST communities. In Ranchi and Pakaur, the tribal population ranges from 41.8% to 44.6%. Kodarma (0.8%) and Chatra (3.8%) have the lowest proportions of STs.

The 32 tribal communities in Jharkhand include Munda, Santhal, Oraon, Kharia, Gond, Kol, Kanwar, Savar, Asur, Baiga, Banjara, Bathudi, Bedia, Binjhia, Birhor, Birjiali, Chero, Chick- Baraik, Gorait, Ho, Karmali, Kharwar, Khond, Kisan, Kora, Korwa, Lohra, Mahli, Mal- Paharia, Parhaiya, Sauria-Paharia, and Bhumij.”(6)

# Literature Review

“SCD is a genetic condition affecting red blood cells, resulting from either inheriting two copies of the hemoglobin S mutation (HbSS) or having one copy of this mutation combined with another β-globin chain abnormality. India has some of the highest frequencies of the hemoglobin S allele globally and ranks third in the number of births with HbSS. This condition is found among various ethnic and geographic groups. Particularly, the βS mutation is notably prevalent among indigenous tribal populations (Adivasis), who represent over 8% of the population, and historically marginalized scheduled castes, accounting for over 10% of the population (208 million out of more than 1.4 billion people), with an estimated prevalence of 1 in 86 births affected by sickle cell disease.”(7). “The sickle cell gene is commonly found in various tribal communities across India, with prevalence rates ranging from 1% to 40%.”(8). “In a retrospective study conducted at the Department of Pathology, RIMS, Ranchi, researchers examined cases of hereditary hemoglobin disorders from July 2018 to February 2020. The study involved individuals who tested positive for these disorders using solubility tests and the Naked Eye Single Tube Red Cell Osmotic Fragility Test (NESTROFT), followed by High-Performance Liquid Chromatography (HPLC BIO-RAD variant-II SYSTEM). Out of 2023 cases that were positive in screening, 341 (16.85%) showed no evidence of hereditary hemoglobin disorders in the HPLC analysis. Among the confirmed cases, 1294 (64%) were diagnosed with sickle cell disorder. The study also found the prevalence of other hereditary hemoglobin disorders: Beta Thalassemia trait was present in 301 (15%) cases, Beta Thalassemia major in 63 (3%) cases, HbF in 16 (0.8%) cases, HbE trait in 3 (0.14%) cases, HbD trait and HbE Beta Thalassemia each in 2 (0.09%) cases, and HbE disease in 1 (0.05%) case.”(9) “Sickle cell disease is especially widespread among tribal communities in India, with a high prevalence across 17 states. A screening initiative led by the Ministry of Tribal Affairs, in collaboration with the ICMR and the Department of

Biotechnology, found that about 10% of the tribal population carries the trait, while the disease itself affects roughly 1% of this group. The condition is also present in various other communities and regions beyond the tribal populations.” (NSCAEM,2023)“SCD is a genetic condition affecting red blood cells, resulting from either inheriting two copies of the hemoglobin S mutation (HbSS) or having one copy of this mutation combined with another β-globin chain abnormality. India has some of the highest frequencies of the hemoglobin S allele globally and ranks third in the number of births with HbSS. This condition is found among various ethnic and geographic groups. Particularly, the βS mutation is notably prevalent among indigenous tribal populations (Adivasis), who represent over 8% of the population, and historically marginalized scheduled castes, accounting for over 10% of the population (208 million out of more than 1.4 billion people), with an estimated prevalence of

1 in 86 births affected by sickle cell disease.” (6). “The sickle cell gene is commonly found in various tribal communities across India, with prevalence rates ranging from 1% to 40%.” (7).

“A study in the Al-Ahsa region of Saudi Arabia assessed Sickle Cell Disease (SCD) awareness among intermediate and high school students, a decade after the introduction of a premarital screening program. The research involved 1,500 students from urban and rural areas, who were surveyed using a questionnaire to gauge their knowledge of SCD. Of the respondents, 79% completed the survey, with a higher proportion of females (62%) compared to males (38%). Most participants were aged 16-19, with a notable number being high school students. Among them, 8.3% had SCD, and 14.3% were unaware of their condition. Overall, 89% of the students had heard of SCD, and those familiar with the disease tended to give more accurate answers. While female students showed slightly better knowledge, this difference was not statistically significant. Age did not significantly impact knowledge levels, although older students generally answered more questions correctly. The study identified several misconceptions about SCD, including its complications and influencing factors.

Although most respondents correctly identified common complications like growth failure, jaundice, bone pain, and abdominal pain, there was limited understanding of the effects of extreme weather, strenuous exercise, and tobacco use on SCD. Furthermore, many were unclear about the impact of fluid intake and diet. While 77.7% of students were aware of the premarital screening program and 85.8% recognized its importance, only 25% thought SCD could be cured, and many were uncertain about whether they had been screened. The findings suggest that although general awareness of SCD and its complications is high, there are significant gaps in knowledge regarding preventive measures and the disease’s overall nature.”(10)

“Sickle Cell Disease is associated with high rates of comorbidities and mortality, yet there is limited research on the quality of life (QOL) for adolescents with SCD or Sickle Cell Trait (SCT). A study conducted in Orissa seeks to address this gap by examining QOL among adolescents in the Koraput district of Odisha, an area with a high prevalence of sickle cell hemoglobinopathies but lacking in extensive population-based research. Using a modified QOL scale and snowball sampling, the study evaluated the physical, psychological, and social well-being of 387 adolescents. Results revealed that those with SCD experienced significantly more hospitalizations and lower QOL scores in all areas compared to adolescents with SCT and healthy peers. Although adolescents with SCT are often asymptomatic, they still face challenges that impact their QOL, such as frequent colds and perceived economic pressures.”(11)

“A study conducted in Kampala City, Uganda, employed an analytical cross-sectional approach to assess the prevalence of sickle cell trait (SCT) and factors affecting screening uptake among secondary school students. Data were gathered through semi structured questionnaires to evaluate students' knowledge, attitudes, and factors influencing SCT screening. Blood samples from consenting participants were analyzed for SCT, with results

processed using SPSS software. The study targeted advanced-level secondary school students in Kampala, involving a total of 399 participants, and all ethical approvals and consents were secured before data collection. The findings revealed that 5.8% of the participants had SCT (HbAS). A significant majority (67%) held a negative attitude towards SCT testing, although 89.7% of those who consented to testing were interested in knowing their sickle cell status.

Only 1.5% had previously undergone sickle cell testing, and most participants demonstrated moderate understanding of sickle cell disease (SCD) and its screening process. Despite being aware of SCD's hereditary nature, many participants had misconceptions, such as the belief that sickle cell carriers frequently experienced medical issues and had shorter lifespans.

Concerns about stigma and discomfort during testing also influenced their willingness to participate. The study found significant correlations between SCT testing and factors such as knowledge of a partner's sickle cell status and religious affiliation (Anglican).”(12)

Solubility Sickling Test – “This test identifies the polymerization of hemoglobin S (HbS) when it is deoxygenated. It is widely used due to its simplicity and affordability. The principle behind the test is that HbS becomes insoluble in a concentrated phosphate buffer mixed with a hemolyzing agent and sodium dithionate. These substances cause HbS to crystallize and precipitate, resulting in a turbid solution due to light scattering. The test outcomes are compared to known negative and positive controls. Despite its ease of use, the solubility test has limitations. It may produce false-negative results in newborns because of high levels of hemoglobin F, or if HbS is less than 10% of the total hemoglobin. False negatives can also occur in individuals with α-thalassemia traits or severe anemia.

Conversely, false positives can happen in cases of high serum viscosity, erythrocytosis, significant leukocytosis, or certain anemias. To reduce the likelihood of missing sickle cell cases, it is advisable to use this test alongside hemoglobin electrophoresis or other hemoglobin assays, which provide a more detailed evaluation of hemoglobin types and

levels. Additionally, the solubility test cannot differentiate between sickle cell trait (SCT) and sickle cell disease (SCD) and is less effective at detecting hemoglobin AS(HbAS).” [25,26]. “These drawbacks limit its effectiveness in screening programs.(13)

A study conducted in the Koraput district of Odisha assessed the knowledge, awareness, and attitudes of individuals concerning premarital genetic counseling and screening for sickle cell hemoglobin. Out of 152 participants, 92.76% were aware of sickle cell anemia, but only 30.26% had knowledge about premarital screening. The findings indicated that understanding of premarital screening for sickle cell disease (SCD) was generally low, with only 30% of patients and their families recognizing its significance. Additionally, participants were more familiar with premarital screening compared to post-marital screening, though overall awareness remained limited, with just 29% reporting any knowledge of premarital screening.Around 32% of respondents believed premarital screening could identify multiple diseases and was not restricted to SCD, while 10% associated it solely with SCD. A majority, approximately 58%, lacked any awareness or opinion on premarital screening. Regarding diseases other than SCD, participants showed higher awareness of HIV (40%) and both HIV and hepatitis (44%). Concerning the effectiveness of premarital screening in preventing SCD, nearly 60% of individuals believed it could help, but 31% were uncertain.In terms of inheritance, 90% of participants believed that carriers could not directly transmit the disease, and 80% agreed that awareness of sickle cell screening could aid in avoiding incompatible partnerships. However, none of the participants knew their genetic profiles, highlighting a lack of genetic-level awareness.Among married individuals, only 7% had undergone any testing prior to marriage. Regarding treatment preferences, 33% of those with SCD relied exclusively on traditional remedies, 42% used both traditional and allopathic treatments, and 26% depended solely on allopathic medicine.(14)

The study examined the awareness of sickle cell disease (SCD) in Chhotaudepur, Gujarat, within its tribal population. A health education initiative was introduced to enhance knowledge. Initially, 75.3% of participants were familiar with SCD, but only 20% knew its cause. Following the intervention, 42% more individuals accurately identified the cause, and 83% recognized pain as a primary symptom. By the study's conclusion, 77% were aware of modern treatment options. These results highlight the critical role of community-centered educational efforts in alleviating the burden of SCD.(15)

# Research Gaps identified

Previous studies have found limitations which include the exclusion of ninth-grade students from intermediate schools, where Mendelian inheritance is not taught, potentially impacting their understanding of SCD. Additionally, the studies with cross-sectional design limit their ability to establish causality or track changes in knowledge over time. A notable research gap is the need for educational interventions tailored to younger students to enhance their understanding of SCD. studies also suggest incorporating SCD awareness into secondary school curricula and continuing public health campaigns to improve knowledge. Furthermore, few other studies suggested neonatal screening programs to enable early detection and intervention for SCD, rather than relying solely on premarital screening.(10)

There is a need for large-scale, population-based studies that cover all regions with high sickle cell prevalence, particularly in underserved areas. There is also a lack of in-depth studies focusing on the QOL of adolescents with sickle cell hemoglobinopathy, especially sickle cell trait (SCT), which is often overlooked due to its relatively asymptomatic nature. Moreover, educational interventions targeting adolescents and their families to improve understanding and management of SCD and SCT are necessary. Further research should

explore the psychosocial impacts and economic burdens on families affected by SCD, emphasizing the need for comprehensive healthcare support and awareness programs.(11)

There is also a gap in understanding the social and cultural barriers that contribute to negative attitudes toward SCT testing. There is also a direct need of developing strategies to address misconceptions and fears related to sickle cell screening, as well as enhancing the accessibility and affordability of testing services. Further research is needed to explore these areas and to develop interventions that can effectively promote SCT screening among young populations.(12) The present study focuses on the research gaps identified through creating awareness among adolescents of tribal areas of east Singhbhum district, Jharkhand along with early detection of SCD for timely intervention for effective health outcomes.

# Objectives

1. Create awareness among adolescents in Government Schools towards sickle cell disease.
   1. Assess the baseline knowledge and attitudes of participants before the Health Education Awareness session.
   2. Assess the effect of awareness sessions on knowledge and attitudes post-session, & three months after the Health Education Awareness session.
   3. Evaluate the feedback of the participants on the awareness session.
   4. Compare the level of awareness and attitude of Scheduled Tribes with non-Scheduled Tribes adolescents in government schools concerning SCD.
2. Conduct school based screening and early detection for anemia and sickle cell disease, followed by counselling and referral among adolescents of the Government School.
   1. Compare the socio-demographic factors of ST with non-ST adolescents concerning Sickle Cell Disease.
3. Evaluate the perceptions of all stakeholders regarding the National Sickle Cell Anemia Elimination Mission(NSCAEM),2023.
   1. Perspective of service providers towards the NSCAEM,2023.
   2. Perspective of beneficiaries towards the NSCAEM ,2023.

# Detailed Methodology

**Research Design: Sequential Explanatory Mixed-Methods Design**

This design involves first collecting and analyzing quantitative data, followed by qualitative data collection to further explain or elaborate on the initial findings.

# Sequential Design

A sequential design refers to the chronological order in which the study's quantitative and qualitative phases are conducted. In this case, the study progresses systematically, with the quantitative phase occurring first, followed by the qualitative phase.

# Quantitative Phase (Initial)

The study begins by administering a structured questionnaire to evaluate adolescents' baseline knowledge and attitudes about Sickle Cell Disease (SCD). This is followed by an educational intervention, such as a health awareness session, to provide information on SCD, its symptoms, prevention, and management strategies. After the intervention, the same questionnaire is re-administered to assess the short-term effects of the education. A follow-up is conducted three months later to evaluate knowledge retention and any changes in attitudes over time. Additionally, a screening is performed concurrently to identify cases of SCD and anemia, collecting data on the adolescents' health status.

# Qualitative Phase (Subsequent)

Once the quantitative data has been gathered and analyzed, the qualitative phase begins. In- depth interviews are conducted with key stakeholders, including healthcare providers, ‘ASHA’(16) workers, caregivers, and individuals affected by SCD, to gain a deeper understanding of the barriers to accessing healthcare, perceptions of SCD, and insights into the National Sickle Cell Anemia Elimination Mission(NSCAEM).

# Explanatory Nature

This design is explanatory because the qualitative phase explains and provides context to the findings from the quantitative phase.

Quantitative Data as the Foundation

The quantitative phase establishes baseline information such as how much adolescents know about SCD, their attitudes towards it, and the rates of SCD and anemia among the adolescents screened. The post-test and three-month follow-up questionnaires provide data on the effectiveness of the educational intervention in enhancing knowledge and shifting attitudes.

Screening data also offers insights into the prevalence of SCD and anemia within the study population.

Qualitative Data for Deeper Insights

The quantitative phase may raise critical questions that require further exploration. For example, if differences in knowledge retention or SCD awareness emerge between specific groups (e.g., Scheduled Tribes vs. Non-Scheduled Tribes), the qualitative interviews can help explain why such disparities exist. Interviews with stakeholders, such as healthcare providers and caregivers, offer valuable insights into obstacles to healthcare access—be they geographic, economic, or cultural—that may have influenced the quantitative results (e.g.,

low participation in screening or limited knowledge retention). The qualitative data allows for a more detailed understanding of the personal experiences of both healthcare providers and patients, which might not be fully captured by quantitative data alone. For example, healthcare workers might report difficulties in reaching remote communities, while caregivers may discuss cultural beliefs that hinder treatment-seeking behaviors.

1. Rationale for a Sequential Explanatory Mixed-Methods Design Understanding Both Outcomes and Context:

The quantitative phase provides a broad, population-level understanding of adolescents' knowledge about SCD and the effectiveness of the intervention. However, it doesn’t explain why these outcomes occurred or how they can be improved. The qualitative phase enriches the study by offering context-specific insights that help explain why some adolescents struggle with knowledge retention, why barriers to screening persist, or how stakeholders perceive the effectiveness of interventions like the National Sickle Cell Anemia Elimination Mission(NSCAEM).

# STUDY POPULATION AND SAMPLING

“The study focuses on adolescents people between the ages of 13 to 19, a crucial developmental stage according to the World Health Organization (WHO).”(17)This age range is selected to capture a diverse range of experiences and knowledge relevant to SCD. Educating adolescents before marriage is intended to empower them with information necessary for informed decisions regarding carrier screening and genetic counselling, potentially reducing SCD prevalence in future generations.

In order to achieve a representative and statistically robust sample for this study, we will implement a Multistage Random Sampling to draw the study population from government schools in the East Singhbhum district of Jharkhand, India.

sa From each school, students between 13 – 19 years will be selected by complete enumeration. This targeted approach is designed to ensure effective follow-up, as students in higher grades are more likely to leave school before the study is completed. By focusing on students in classes 7, 8 ,9,10,11and 12 the study aims to maintain a stable cohort throughout the research period, thereby enhancing the reliability and consistency of longitudinal data collection. This comprehensive screening process ensures that no eligible adolescent is excluded and helps to minimize sampling bias.

Additionally, the study will form separate groups for Scheduled Tribes and Non-Scheduled Tribes to explore any specific differences in socio-demographic factors, their level of awareness and attitude in terms of sickle cell disease.

Stakeholder Sampling for qualitative data: Convenience sampling will be used for key stakeholders like healthcare Providers, Caregivers, Educators, and health care workers.

# INCLUSION CRITERIA

1. Participants enrolled in that Government school
2. Participants who gave assent for the study and received consent from their guardians.
3. Participants should be within the adolescent age range between 13 to 19 years old.

# EXCLUSION CRITERIA

1. Participants who are planning to transfer to another school before the study concludes.
2. Those participants who were absent on any of the visits.

# SAMPLE SIZE

**Quantitative Phase:**

n = Z2⋅p⋅(1−p) / d2 (Leslie Fischer’s Formula) Z represents the confidence level (95%)

P is the prevalence of sickle cell trait (10%) q equals 1-p

d denotes the margin of error (5%)

Initially, the sample size is 139. After accounting for a design effect of 2, the adjusted sample size becomes 278

To account for a 20% attrition rate, we need to increase the sample size.

After including an attrition of 20%, the Final Sample size = 347.5, Rounding up to 360

# Qualitative Phase:

**Healthcare Providers** Medical Officers and Doctors Nurses and Healthcare Staff **Community Health Workers**

Accredited Social Health Activists (ASHAs) Multi-Purpose Workers (MPWs)

# Educators

School Principals

Teachers

# Caregivers of SCD Patients

Family members or caregivers of SCD patients

**Adolescent Research Participants identified with Sickle Cell Disease** Adolescent Research Participants diagnosed with sickle cell disease **Total Sample Size – 60**

# Data will continue to be gathered until saturation is reached. This means the collection will proceed until no new themes or insights are emerging from the interviews of each stakeholders.

**DATA COLLECTION METHODS:**

**Structured Questionnaire:** Participants will complete a structured questionnaire which is focused at assessing their knowledge and attitudes regarding sickle cell disease. This tool will measure awareness, understanding of the disease's implications, and perceptions about its management and prevention and their attitude towards Sickle Cell Disease.

**Participants Screening:** The screening process will be conducted in two phases.

Hemoglobin Level Assessment and Sickle Cell Disease Screening: Comprehensive Procedure

# Hemoglobin Level Assessment

Objective: The hemoglobin level assessment aims to evaluate the overall condition of anemia in participants. Anemia, if present, may complicate the identification of sickle cell disease (SCD) and affect diagnostic precision.

Location and Setting:

Site Preparation:

Venue: Hemoglobin level assessments will be carried out in designated areas within the participating schools. These areas will be designated as temporary blood collection and processing stations.

Setup: Tables and chairs will be arranged to create a clean and organized space. Blood collection and processing areas will be separated to maintain hygiene and avoid cross- contamination.

Equipment and Supplies:

Sample Collection: Finger Prick Instruments:

Lancets: Sterile, single-use lancets designed for blood collection. Capillary Tubes: For collecting the blood droplets from the finger prick. Alcohol Swabs: To disinfect the skin before needle insertion.

Bandages: For post-collection care. Analysis:

Hemoglobin Meters: Portable hemoglobin meters will be used for point-of-care testing. Personal Protective Equipment (PPE):

Gloves: Sterile gloves to prevent contamination.

Masks: To reduce the risk of respiratory droplet transmission.

Lab Coats: To protect clothing and maintain a clean environment. Procedure:

Participant Preparation:

Explanation and Consent: Participants will be informed about the procedure's purpose, including how the hemoglobin test helps identify anemia and its impact on SCD diagnostics. Written consent will be obtained from guardians will be taken.

Preparation for Collection: Participants will be seated comfortably. Sample Collection:

Disinfection: The selected area will be cleaned with an alcohol swab and allowed to dry.

Blood Draw: The phlebotomist / Lab Technician will prick one of the finger, collect the blood . Pressure will be applied to the site to prevent bleeding.

Post-Collection Care: A bandage will be applied to the puncture site, and the participant will be monitored briefly to ensure no immediate adverse reactions.

Hemoglobin Measurement:

Immediate Processing:

Hemoglobin Meter: The blood sample on a test strip will be inserted into the portable hemoglobin meter. The device will provide a reading within minutes.

Assessment Criteria:

Normal Hemoglobin Levels: 12.0 - 15.5 g/dL for adolescents. Anemia Classification:

Mild Anemia: Hemoglobin levels between 10.0 - 11.9 g/dL. Moderate Anemia: Hemoglobin levels between 7.0 - 9.9 g/dL. Severe Anemia: Hemoglobin levels below 7.0 g/dL.

Documentation: Results will be recorded on individual participant forms and stored securely. Quality Control and Safety:

Waste Disposal:

Biohazard Bags: Used needles, tubes, and swabs will be disposed of in red biohazard bags. Sharps Containers: Used needles will be placed in puncture-resistant sharps containers.

Sample Handling:

Labelling: Strip will be labelled with participant identifiers and stored. Hygiene: Regular handwashing and disinfection practices will be followed.

# Sickle Cell Disease Screening

Objective: To identify the presence of sickle cell hemoglobin (HbS) using a solubility test. This screening helps determine if further confirmatory tests are needed.

Location and Setting:

Testing Facilities:

Lab Setup: The solubility test will be conducted in a mobile lab unit set up at the school. The lab area will be equipped with the necessary reagents and equipment.

Hygiene: The testing area will be cleaned and disinfected before and after each testing session to maintain a sterile environment.

Equipment and Supplies:

Solubility Test Reagents: Prepared according to the manufacturer's instructions. These include solutions that react with sickle hemoglobin to form a precipitate.

Testing Apparatus: Test tubes, pipettes, and mixing equipment for the solubility reaction.

Personal Protective Equipment: Gloves, lab coats, and face shields or goggles to protect staff and maintain a clean environment.

Procedure:

Sample Preparation:

Reagent Preparation: Reagents will be prepared by mixing according to the provided protocol. They will be stored at the recommended temperature and used before their expiry date.

Blood Sample Handling: Phlebotomist / Lab Technician will ensure safe handling of blood samples collected from participants and it will be mixed with a prepared reagent in a test tube.

Solubility Test Execution:

Mixing: A measured volume of blood (usually20 micro litre) will be added to the reagent in the test tube. The solution will be mixed thoroughly.

Incubation: The test tube will be incubated at a controlled temperature (usually around 37°C) for 5-10 minutes.

Observation:

Positive Result: Cloudiness or turbidity in the solution indicates the presence of sickle hemoglobin (HbS). The solution will appear turbid compared to a control sample.

Negative Result: The solution remains clear, indicating the absence of sickle hemoglobin.

Documentation: Test results will be recorded on a results sheet, noting any abnormalities or positive findings.

Post-Test Procedures:

Result Communication:

Notification: Participants will be informed of their test results, either directly or through guardians. The communication will include explanations and next steps.

Counselling: Participants with positive SCD results will receive counseling, including information on the condition, potential management options, and support resources.

Referral and Follow-Up:

Referral: Participants requiring further evaluation or treatment will be referred to specialized clinics or healthcare providers.

Follow-Up: A system will be established to track and follow up with participants for additional testing or support services if necessary.

This detailed procedure ensures that both the hemoglobin level assessment and SCD screening are conducted thoroughly, accurately, and ethically, providing valuable information for the management and understanding of anemia and sickle cell disease in the study population.

Hemoglobin Level Assessment: The first phase involves testing hemoglobin levels to evaluate the general condition of anemia. This step is essential because severe anemia might complicate the identification of sickle cell disease and impact the precision of the diagnosis.

Sickle Cell Disease: After the initial evaluation, a solubility test will be used to identify the presence of sickle cell hemoglobin..

In-depth Interviews: To gain insights into barriers and perceptions related to sickle cell disease, we will conduct in-depth interviews. These interviews will explore participants' experiences, challenges faced in accessing healthcare services, and their views on sickle cell

disease management and prevention. This will include both service provider’s as well as service receivers’ perceptions.

# DATA COLLECTION PROCEDURE

1. **Approval and Permissions**

Institutional Ethics Committee (IEC) Approval:

Before the commencement of the study, approval will be sought from the Institutional Ethics Committee (IEC). This step ensures that the study complies with ethical standards and that participants' rights are protected.A detailed research proposal will be submitted to the IEC, covering the study's purpose, procedures, potential risks, and benefits.

The proposal will emphasize the importance of maintaining participant confidentiality, ensuring that sensitive medical data (e.g., test results) is securely stored and only accessed by authorized personnel.

The study will ensure that participants understand their right to withdraw from the study at any point, without any negative consequences.

District Educational Officer (DEO) Permission:

A formal letter will be drafted and sent to the District Educational Officer (DEO), detailing the study's objectives, its significance for adolescent health, and the need for permission to access government schools.

The letter will highlight the importance of sickle cell disease (SCD) screening in the adolescent age group, especially for early detection and intervention in regions where the disease is prevalent.

Meetings will be scheduled with the DEO to discuss the study plan, timelines, and any school-related protocols that need to be followed.

State Tribal Welfare Department Approval:

For schools located in tribal areas, approval will be obtained from the State Tribal Welfare Department to ensure compliance with local governmental policies and healthcare initiatives.

Civil surgeons, local tribal leaders, and community representatives will be informed about the study to secure community participation and engagement.

The importance of screening for sickle cell disease in tribal populations, where the disease burden is higher, will be emphasized to ensure the smooth execution of the study in these regions.

# Permission from School Principals

Principal Consent:

School principals will be approached through formal letters, requesting permission to conduct the study on their premises. The letter will outline the study’s objectives, methodology, and potential benefits for students and their families.

Meetings will be scheduled with school principals and administrators to explain the study in detail, including how the data will be collected, the role of the school in facilitating logistics, and the importance of awareness and screening for SCD.

Principals will be required to provide formal written consent, confirming their approval to allow access to students for the educational sessions, questionnaires, and blood sample collection.

Any special considerations for the timing of sessions (e.g., during specific school hours to minimize disruption) will be discussed and agreed upon.

# Logistics and Precautions

Coordination with Lab Technician/Phlebotomist:

A certified Lab Technician/phlebotomist, experienced in adolescent blood sample collection, will accompany the research team. Their role is crucial for ensuring that blood samples are collected safely and efficiently from participants.

All required equipment, including sterile syringes, vacutainers, alcohol swabs, sterile gauze, band-aids, and sample collection tubes, will be organized before visiting each school. Each item will be carefully checked for expiration and sterility. A calm and friendly demeanor will be maintained to ensure the students feel comfortable during the blood collection process.

Bio-Medical Waste Management:

**Red biohazard bags** will be used on-site to collect and safely store any biological waste generated during the blood collection process. These include used syringes, gauze, cotton balls, gloves, and other disposable medical supplies.

**A sharp waste disposal container**, compliant with biomedical waste disposal regulations, will be provided specifically for used needles and other sharp objects to prevent accidental injuries.

**Personal Protective Equipment (PPE)**, such as gloves, face masks, and apron, will be worn by the lab technician/phlebotomist involved in the sample collection process. PPE will be discarded in sealed biohazard bags after each session.

All biohazard waste will be securely transported in sealed containers back to an authorized biomedical waste management facility with help of Manipal Tata Medical College, ensuring proper disposal as per government regulations. Special care will be taken to follow local protocols for handling and disposing of medical waste in schools.

# Initial Briefing and Educational Session

Introduction to Sickle Cell Disease:

Upon arrival at each school, the research team will conduct a short educational session to introduce students to sickle cell disease (SCD). This session will include a brief, age- appropriate explanation of the genetic nature of the disease, its common symptoms (e.g., pain crises, anemia), and why early detection is critical.

The session will be designed to engage students through interactive discussions and encourage questions, with a focus on alleviating any fears or concerns they may have about the screening process.

# Engagement Session:

Visual aids such as posters, diagrams, and PowerPoint presentations will be used to make the session engaging and easy to understand. The materials will be customized for the local context, ensuring they are relatable and culturally appropriate.

The research team will use simple language to explain complex genetic concepts, making sure all students, regardless of their educational background, can follow the discussion.

# Informed Consent and Assent

Assent & Consent Process:

Before any screening or testing begins, written informed consent will be obtained from the parents or legal guardians of all participants. A detailed information sheet will be provided, outlining the study’s objectives, procedures, potential risks, and benefits.

The consent form will clearly state that participation is voluntary and that there are no penalties for withdrawing from the study at any point.

Participants themselves will provide assent, especially since they are adolescents. The assent form will be written in simple, understandable language, ensuring they fully comprehend what their involvement entails.

Research staff will be available to answer any questions from parents or students, ensuring that everyone involved understands the process and the purpose of the study.

In this research study , two separate sets of consent and assent forms will be obtained from the research participants:

**Consent and Assent During Quantitative Data Collection and Health Awareness Session:** Before the initial phase of the research, which involves quantitative data collection and the health awareness session, written informed consent will be obtained from the guardians or parents of the adolescents who are selected in our research study. At the same time, an assent form will be provided for the adolescents themselves, ensuring that they fully understand the purpose of the study and agree to participate. This will allow the adolescents to participate in completing the baseline questionnaire and attending the educational session.

**Consent and Assent for the Screening Phase (Hb Level Estimation and Sickle Cell Detection):** Before the screening phase, which includes Hb level estimation and detection of sickle cell disease, a second set of consent and assent will be obtained. Guardians will be

asked to provide informed consent for their adolescent children to participate in the screening process. Adolescents will also give their assent, acknowledging their understanding and willingness to undergo the screening tests. This step ensures that both the legal guardians and the participants are fully informed about the procedures and provide their permission specifically for the medical screening.

Consent Storage:

All signed consent and assent forms will be collected, and securely stored in locked filing cabinets, and digital copies will be encrypted. Access to these forms will be restricted to the research team members responsible for maintaining confidentiality.

# Knowledge and Attitude Assessment

Questionnaire Development:

A Knowledge and Attitude questionnaire will be developed based on a thorough review of the literature and validated by a panel of experts. This questionnaire will assess the participants’ baseline knowledge of sickle cell disease, their attitudes toward genetic screening, and any misconceptions they may have.

The questionnaire will include sections on common symptoms, modes of inheritance, treatment options, and preventive measures. It will also evaluate students' understanding of the impact of SCD on daily life.

Questionnaire Administration:

Before the educational session, participants will fill out the validated questionnaire. Research team member will be available to assist students if they have any questions or difficulties understanding the questionnaire.

The baseline data collected from the questionnaire will be used to measure the effectiveness of the educational intervention by comparing it to the post-education questionnaire results.

# Awareness and Educational Intervention Educational Session:

“A health education intervention is a planned approach aimed at enhancing people's awareness and understanding to inspire changes in their health behaviors, with the primary objective of disease prevention or improving overall well-being and better health outcomes. This process involves informing individuals or groups about various health issues, including disease causes, preventive strategies, and adherence to treatments. The scope of these interventions can range from sharing basic to more comprehensive efforts aimed at promoting lifestyle adjustments. Successful health education initiatives not only focus on imparting knowledge but also consider people's ability, readiness, and motivation to act on that information in their daily routines.”(18)

After completing the baseline questionnaire assessment, a more detailed educational session will be held. This session will be between 45 minutes to 1 hour, ensuring there is ample time to cover all the necessary topics without overwhelming the participants. The session will conclude with a Question & Answer segment, allowing students to ask questions and clarify their doubts. This interactive portion will further reinforce their understanding. The session will focus on providing in-depth information about SCD, including:

Symptoms: Explaining common symptoms like severe pain, frequent infections, and delayed growth.

Genetic Transmission: Explaining how SCD is passed from parents to children, using easy- to-understand diagrams of inheritance patterns.

Importance of Screening: Emphasizing the role of early detection in preventing complications and offering examples of treatment options available.

IEC Materials:

Information, Education, and Communication (IEC) materials such as leaflets, brochures, and posters will be distributed to students. These materials will be customized to the local context and printed in both English and the local language.

The materials will include contact information for local healthcare providers and resources for families who may need further assistance.

In total 12 health awareness sessions will be conducted to effectively reach all 350 participants in my research study. Each session will include 30 participants because I believe this group size is ideal for fostering engagement and encouraging participation during the session. With 30 participants, I can ensure that everyone can ask questions, clarify doubts, and fully engage with the material. By holding 12 sessions, I can accommodate all 350 participants. The calculation is simple: dividing 350 by 30 participants per session results in about 11.67, so I will round this up to 12 sessions. This approach will allow me to maintain the effectiveness of each Health Awareness session while ensuring that every individual receives the attention and information they need.

# Post-Test Assessment and Feedback

Post-Educational Questionnaire:

After the educational session, participants will be given the same questionnaire to assess how much their knowledge and attitudes toward SCD have changed.

Data from this post-test will be compared to the baseline results to measure the immediate impact of the educational intervention.

Feedback:

A feedback form will be distributed to students to evaluate their experience with the session. The form will ask about the clarity of the materials presented, the usefulness of the information provided, and whether they feel better informed about SCD.

# Follow-Up Assessment

3-Month Knowledge Retention Test:

Three months after the initial session, a follow-up visit will be conducted, during which the same questionnaire will be administered again. This will measure how well the students retained the information over time.

The retention test will provide insight into the long-term effectiveness of the educational intervention and identify areas that may require reinforcement.

# Data Analysis

Statistical Analysis:

All collected data, including knowledge scores from the Knowledge and Attitude questionnaires, prevalence of sickle cell disease, and demographic information, will be analyzed using statistical software.

Descriptive statistics will summarize the prevalence of SCD among the participants, while inferential statistics will be used to compare pre- and post-education knowledge scores and assess the effectiveness of the intervention.

Additional analysis will explore correlations between knowledge levels and other variables such as gender, socio-economic status, and family history of SCD.

During the analysis phase,will focus on drawing a meaningful correlation between hemoglobin (Hb) levels and the presence of sickle cell disease (SCD) among the study population who are adolescents of the Government schools . Hemoglobin levels are a critical clinical indicator, as they reflect the oxygen-carrying capacity of red blood cells, which is often compromised in individuals with sickle cell disease.In sickle cell patients, the abnormal hemoglobin (HbS) causes red blood cells to deform into a sickle shape, leading to hemolysis and a reduction in overall hemoglobin levels. By analyzing Hb levels, we aim to uncover patterns that correlate with the clinical manifestations of SCD. This correlation could reveal whether lower Hb levels are associated with more frequent or severe complications, such as painful crises, anemia, or susceptibility to infections.

Using statistical methods, we will compare the hemoglobin values across individuals diagnosed with sickle cell disease and those without, which will enable us to identify trends that might indicate the degree to which Hb levels predict the presence of sickle cell disease.

# Stakeholder Perception Evaluation

In-Depth Interviews with Key Stakeholders:

To gain a comprehensive understanding of the perceptions surrounding the National Sickle Cell Anemia Elimination Mission(NSCAEM) and sickle cell disease (SCD) management strategies, in-depth interviews will be conducted with a diverse range of stakeholders. These stakeholders include medical officers, nurses, Accredited Social Health Activists (ASHAs)(16), Multi-Purpose Workers (MPWs), SCD patients, Students , parents of the students and Principals. These individuals play a crucial role in both the implementation of the program and the day-to-day management of SCD at various levels, making their insights vital for assessing the program's efficacy and areas for improvement.

Stakeholder Groups:

Medical Officers and Healthcare Providers:

Medical officers and doctors working at local government health centers, hospitals, or SCD clinics will be interviewed to assess their understanding of the program’s objectives, their involvement in SCD management, and the challenges they face in delivering effective healthcare services.

Focus will be placed on their experience with NSCAEM's infrastructure, support systems, availability of necessary resources (such as medications like hydroxyurea), and their observations on patient outcomes since the program’s implementation.

Nurses and Healthcare Staff:

Nurses and other healthcare personnel who interact directly with SCD patients during routine checkups or hospital visits will provide insights into their hands-on experience with patient care, medication administration, and follow-up protocols.

Interviews with this group will focus on identifying gaps in training, support, or resources that may hinder the delivery of quality care, as well as any perceived improvements in patient outcomes due to the NSCAEM.

Accredited Social Health Activists (ASHAs)(16):

ASHA workers, who often serve as the first point of contact between rural communities and the healthcare system, will provide critical insights into the program's community-level implementation. Their role in conducting household visits, health education, and patient monitoring is invaluable in early detection and ensuring treatment adherence.

ASHA workers will be asked about their knowledge of SCD, the challenges they face in educating families about the disease, and their involvement in encouraging families to

participate in screening programs. They will also share their experiences in following up with patients, particularly in remote areas where access to healthcare is limited.

“Multi-Purpose Workers (MPWs)”(19):

MPWs, responsible for coordinating public health services, including vaccination and disease surveillance, will provide insights into their experiences in coordinating SCD screening and follow-up services. Their understanding of the program's logistics, coordination between healthcare facilities, and the integration of SCD management with other healthcare services will be explored.

Interviews with MPWs will focus on the effectiveness of communication and collaboration between healthcare workers, particularly in tribal or underserved areas, and how these dynamics affect the success of the NSCAEM.

Sickle Cell Disease Patients:

Patients diagnosed with sickle cell disease will be interviewed to understand their firsthand experiences with the healthcare system, particularly their access to diagnosis, treatment, and ongoing care.

The focus will be on their perception of the NSCAEM’s impact on their health outcomes, the availability and affordability of treatments, and any difficulties they encounter in adhering to prescribed therapies. Interviews will also explore how well patients understand their condition and the importance of regular follow-up care.

Caregivers of SCD Patients:

Caregivers, often family members, play a critical role in the daily management of SCD patients, particularly children. They will be interviewed to gain insights into the challenges

they face in supporting patients with SCD, such as arranging regular checkups, accessing medication, and dealing with complications of the disease.

Caregivers will be asked about their involvement in the NSCAEM, their level of awareness regarding available resources, and any barriers they encounter in navigating the healthcare system.

School Principals and Teachers:

Principals and teachers will provide insights into their experiences with implementing school- based health programs, including SCD awareness and screening. They will offer perspectives on the effectiveness of these programs, challenges faced, and suggestions for improvement.

Interview Focus:

The in-depth interviews will aim to gather qualitative data that reveals the following aspects: Perceptions of the NSCAEM:

Stakeholders will be asked about their understanding of the NSCAEM’s goals and their role in its implementation.

Medical officers and healthcare workers will be questioned on how well they feel the program has been integrated into their existing healthcare duties, whether the resources provided under the program meet the needs of the population, and any improvements in patient care they have observed since its inception.

SCD patients and their caregivers will provide feedback on whether they have seen improvements in access to screening, treatment options, or overall disease management since the program was introduced.

Barriers to Effective Program Implementation:

A key focus of the interviews will be identifying the barriers that stakeholders encounter in implementing the NSCAEM. These may include logistical challenges, such as delays in receiving medications or difficulty in accessing rural populations for screening and follow- up.

Healthcare workers will be asked about shortages of personnel or resources, lack of training on SCD management, or difficulties in coordinating services between different levels of care (e.g., primary, secondary, and tertiary healthcare facilities).

For patients and caregivers, potential barriers might include the cost of traveling to health facilities, fear or stigma associated with SCD, or misunderstandings about the disease and its treatment.

Challenges in Healthcare Access:

Stakeholders will be questioned about the specific challenges faced by patients in accessing healthcare services, especially in tribal or remote areas. This will include examining geographic barriers, transportation difficulties, and the availability of local health centers capable of managing SCD.

Interviews with ASHAs and MPWs will explore the difficulties in reaching high-risk populations, ensuring follow-up for patients who test positive for SCD, and providing adequate education about the disease.

Cultural and Social Challenges:

ASHA workers and caregivers will be asked about any cultural or social factors that may affect the success of the NSCAEM. This could include misconceptions about SCD, stigma associated with the disease, or reluctance to seek medical help.

Patients and their families may face resistance from the community regarding genetic screening or treatment adherence. Understanding these cultural challenges will be crucial for developing more effective awareness campaigns.

Potential Areas of Improvement:

The interviews will also focus on identifying suggestions for improving the program’s implementation. Stakeholders will be encouraged to propose practical solutions based on their experience, such as more frequent training sessions for healthcare workers, better access to diagnostic facilities, or enhanced community outreach efforts.

SCD patients and their caregivers will be asked about what additional support they would like to see, whether in terms of medical care, educational resources, or community support networks.

Stakeholder Collaboration and Communication:

Understanding how well stakeholders at different levels communicate and collaborate will be a critical aspect of the interview process. This includes the coordination between healthcare providers, ASHA workers, MPWs, and local healthcare facilities, which can significantly influence the success of the NSCAEM.

Stakeholders will be asked about the existing channels of communication, the efficiency of referral systems for SCD patients, and the level of support they receive from higher-level health authorities.

Interview Format and Methodology:

Location and Setting:

The In-Depth interviews (IDIs) will be conducted in locations convenient for the participants, ensuring privacy and comfort. Common settings include healthcare centers, community halls,

or participants' homes for caregivers and patients. This will be decided based on participant availability and preference.

Interview Process:

Each interview will follow a semi-structured format with open-ended questions to allow for flexibility and detailed responses. Interviews will be one-on-one to ensure participants can share their experiences freely without external pressure.

Data Collection Methods:

Audio Recording: With participant consent, all interviews will be audio recorded to ensure accurate data collection. This will allow the interviewer to focus on the conversation and follow up on important points without being distracted by extensive note-taking.

Note-Taking: During the interviews, brief notes will be taken to capture any non-verbal cues, significant quotes, and important themes as they arise. These notes will complement the audio recordings.

Duration of Interviews:

Each interview will last approximately 30–60 minutes, depending on the stakeholder’s role and the depth of information provided. Flexibility will be maintained to allow participants to fully express their views.

Participant Consent and Confidentiality:

Before starting the interviews, participants will be informed of the purpose of the study and their role in it. Informed consent will be obtained, ensuring they understand their participation is voluntary, and their responses will remain confidential.

All identifying information will be anonymized during transcription and analysis to maintain participant privacy.

Timeframe:

A timeframe of 6 months is allocated for conducting the IDIs, allowing sufficient time for data collection, transcription, and preliminary analysis.

Data Saturation:

Interviews will continue until data saturation is reached, meaning no new themes or insights are emerging from additional interviews. This ensures that the data collected is comprehensive and representative of the stakeholder perspectives.

Translation and Transcription:

If interviews are conducted in local languages, they will be translated and transcribed into English for analysis.

Data Analysis:

Once the in depth interviews are transcribed, the analysis will begin with a **Phenomenological approach, followed by a Thematic analysis.** Initially, phenomenology will be employed to deeply explore the participants' lived experiences, aiming to uncover their perceptions and interactions with the National Sickle Cell Anemia Elimination Mission(NSCAEM). This method is particularly valuable because it emphasizes how individuals interpret their personal experiences, which is essential for understanding the varied ways in which stakeholders view the implementation and outcomes of Sickle Cell Disease (SCD) management efforts.

After collecting these phenomenological insights, the data will undergo thematic analysis. This step will involve systematically identifying and coding recurring themes, particularly

those related to the program’s implementation, challenges, and the suggestions offered by stakeholders. By using thematic analysis, the data will be organized into structured themes, helping to identify both the issues raised and the solutions recommended by participants.

In addition, a comparative analysis will be carried out. The responses from different groups, such as healthcare professionals,care givers, Principals ,patients, and community members, will be compared to find common themes and differing viewpoints. This process will be critical for identifying areas where the perspectives of healthcare providers and patients on the effectiveness of NSCAEM may not align.

By combining phenomenology and thematic analysis, this approach will provide a holistic understanding of how the NSCAEM and SCD management strategies are both perceived and executed. The findings will shed light on the practical barriers, challenges in collaboration, and socio-cultural influences that shape the SCD management experience. These insights will not only help refine healthcare delivery practices but will also contribute to improving the overall implementation of interventions. Ultimately, the results will be integrated into the broader study on SCD management and used to inform future policy and program decisions aimed at addressing the obstacles in SCD care more effectively.

# Expected outcome Immediate outcome

1. Baseline Knowledge Attitude assessment of Adolescents on SCD.
2. Increased Awareness of SCD among adolescents
3. Early detection of SCD & anemia among adolescents
4. Identification of specific needs and disparities among tribal non-tribal groups of adolescents
5. Development of IEC materials for providing health education in specific groups.
6. Provision of insights into the program's implementation, effectiveness, and areas for improvement from service delivery and receivers’ perspective
7. Comprehensive Understanding of Barriers not related to the program.
8. Generation of a representative sample of data on adolescents with respect to SCD in the form of publication and copyrighting the IEC material.

# Future Scope

1. Reduction of SCD by tailor-made counselling based on participants screening results.
2. Inclusion of awareness sessions as a part of the school curriculum of Jharkhand.
3. Evidence-based recommendations to the policymakers for optimizing the effectiveness of current interventions and enhancing support for affected individuals and communities.
4. Development of strategic interventions through the identification of specific needs and disparities among tribal and non-tribal groups related to sickle cell disease.

# Importance of proposed research investigation and mention the link to relevant SDG Significance of the Proposed Research Study

The planned study on sickle cell disease among adolescents in East Singhbhum district is highly valuable for various reasons:

Early Identification and Care: Sickle cell anemia is a severe genetic condition with significant health and lifestyle implications. Early identification through widespread screening is essential for starting prompt treatment and management. By detecting cases early, the research seeks to lessen illness and enhance overall health results for those affected.

Enhanced Awareness and Education: Raising awareness about sickle cell disease among adolescents is crucial. A significant number of them may not be well-informed about the disease or its consequences. Offering specific educational efforts can enhance their understanding, promote early detection, and aid in making well-informed choices, especially concerning carrier screening and genetic counseling.

Addressing Socio-Demographic Disparities: By comparing socio-demographic factors between Scheduled Tribes (ST) and Non-Scheduled Tribes (Non-ST) adolescents, the research seeks to identify specific needs and disparities. This information can lead to more equitable healthcare solutions and tailored interventions that address the unique challenges faced by different community groups.

Evaluating Program Effectiveness: The investigation will assess the perceptions of various stakeholders regarding the National Sickle Cell Anemia Elimination Mission(NSCAEM). Understanding stakeholder feedback will help refine the program's implementation, enhance its effectiveness, and ensure it meets the needs of the target population.

Informing Policy and Practice: The mixed-methods approach will provide comprehensive data on knowledge, attitudes, and barriers related to sickle cell disease. This evidence will support informed decision-making and policy development, leading to improved healthcare strategies, better resource allocation, and more effective public health interventions.

Improving Healthcare Access: Identifying barriers to accessing healthcare services will help develop strategies to overcome these obstacles, thereby improving the delivery of care and support for individuals with sickle cell disease. This can lead to better health outcomes and reduced healthcare inequities.

# Link to Relevant Sustainable Development Goals (SDGs)(20) SDG 3: Good Health and Well-being(20)

This study aligns with SDG 3, which strives to ensure health and well-being for individuals of all ages. By emphasizing the early identification and treatment of sickle cell disease, the research aids in decreasing mortality rates and enhancing health results. Additionally, the educational aspect of the study supports initiatives to offer universal health coverage and access to essential health services of high quality.

# SDG 4: Quality Education(20)

The study's emphasis on educating adolescents about sickle cell disease supports SDG 4, which focuses on ensuring inclusive and equitable quality education and promoting lifelong learning opportunities. Providing accurate information and raising awareness about the disease enhances the educational experience and empowers adolescents

# SDG 5: Gender Equality(20)

While the primary focus of the research is on sickle cell disease, gender equality is an important aspect to consider. The research can identify any gender-based disparities in knowledge, attitudes, and access to healthcare for sickle cell disease among adolescents. Ensuring both male and female adolescents benefit equally from the study's interventions supports gender equality by addressing any specific needs or barriers faced by different genders. This method ensures fair access to health education and services, enabling all adolescents to make well-informed choices about their health.

# SDG 10: Reduced Inequalities(20)

This research addresses health inequalities by exploring socio-demographic disparities between ST and Non-ST adolescents. By identifying and addressing these disparities, the

study contributes to reducing inequalities in health outcomes and access to healthcare services.

# SDG 17: Partnerships for the Goals(20)

The research involves collaboration with various stakeholders, including healthcare providers, educators, and community workers. This collaborative approach aligns with SDG 17, which highlights the significance of collaboration and partnerships in achieving sustainable development goals. The study's findings can inform and strengthen partnerships aimed at improving health outcomes and program effectiveness.

In summary, the proposed research investigation is crucial for advancing health and well- being, addressing inequalities, and supporting quality education. Its alignment with relevant SDGs underscores its potential impact on public health and social equity.

# Research Time plan

| **Research Activity** | **Time in Months (from Registration)** | | | | | | |
| --- | --- | --- | --- | --- | --- | --- | --- |
|  | 06  (July- January,2025) | 12  (January- July, 2025) | 18  (July- January  ,2026) | 24  (January- July, 2026) | 30  (July- January 2027) | 36  (January- July, 2027) | 42  (July- January 2028) |
| **Course Work** |  |  |  |  |  |  |  |
| **Literature Survey** |  |  |  |  |  |  |  |
| **IEC Approval &**  **Permissions** |  |  |  |  |  |  |  |
| **Scoping Review** |  |  |  |  |  |  |  |
| **Pretest for Knowledge &**  **Attitude Assessment** |  |  |  |  |  |  |  |
| **Post Test for Knowledge**  **& Attitude Assessment** |  |  |  |  |  |  |  |
| **Follow up after 3 months for Knowledge & Attitude**  **Assessment** |  |  |  |  |  |  |  |
| **Participants Screening for Hemoglobin level and**  **sickle cell test** |  |  |  |  |  |  |  |
| **In-Depth Interviews** |  |  |  |  |  |  |  |
| **Data Compilation** |  |  |  |  |  |  |  |

| **DAC presentation and**  **registration renewal** |  |  |  |  |  |  |  |
| --- | --- | --- | --- | --- | --- | --- | --- |
| **Conference presentations** |  |  | **C** |  | **C** |  |  |
| **Journal Publications** |  | **J** |  | **J** |  |  |  |
| **Preparation of Final**  **Thesis** |  |  |  |  |  |  |  |
| **Synopsis presentation** |  |  |  |  |  |  |  |
| **Submission of Thesis** |  |  |  |  |  |  |  |

1. **Pilot study / Preliminary work done Study Setting -** Middle School Ghorabandha **Location –** Ghorabandha

# Overview and Participation

The pilot study was conducted at a Government School in East Singhbhum District. Middle School Ghorabandha was selected for this pilot study focusing specifically on assessing knowledge and attitudes towards sickle cell disease. The study targeted participants from two sections of one Class, that is Class VIIIA and VIIIB. Our aim was to understand their baseline awareness and attitudes, and then measuring the effect of health educational awareness session.

A total of 41 participants, comprising of 16 males (39%) and 25 females (61%) were involved in this study. The program unfolded over 3 days, structured as follows:

# Day 1

Consent forms were distributed to participants and their guardians, alongside assent forms for the students. Ethical compliance was a cornerstone, ensuring that all participants were well informed about the study’s objectives and procedures.

# Day 2

A pre-test questionnaire was administered to gauge the initial or baseline knowledge and attitude of participants towards sickle cell disease. This data served as a critical baseline for subsequent analysis.

# Day 3

A health awareness session was conducted which was solely dedicated to Sickle cell disease. The session included interactive activities and educational material aimed at improving understanding and fostering positive attitudes.

Following this, participants were administered the same questionnaire again, enabling a direct comparison between Pre-Intervention responses and post-intervention responses.

This structured approach ensured a robust evaluation of the health educational intervention’s effect.

**Gender and Class Distribution**

| **Cross Tabulation between Class & Gender** | | | | |
| --- | --- | --- | --- | --- |
| **Class** | | **Gender** | | **Total** |
|  |  |  | |  |
|  |  | **Male** | **Female** |  |
| 1  2 | VIIIA | 9 | 18 | 27 |
|  | VIIIB | 7 | 7 | 14 |
| Total | | 16 | 25 | 41 |

| **Gender Distribution** | | | | | | |
| --- | --- | --- | --- | --- | --- | --- |
| **Gender** | **Frequency** | | **Percent** | | **Cumulative Percent** | |
| Male | | 16 | | 39.0 | | 39.0 |
| Female | | 25 | | 61.0 | | 100.0 |
| Total | | 41 | | 100.0 | |  |

The participant distribution across classes VIIIA and VIIIB offered key insights into

gender representation. Class VIIIA included 27 students, with 9 males and 18 females, while Class VIIIB consisted of 14 students, equally split between males and females. This resulted in an overall female majority, with 61% of participants being female and 39% male.

The higher female participation rate in Class VIIIA reflects demographic characteristics that could influence the study’s outcomes. Understanding this gender imbalance is crucial for interpreting shifts in knowledge and attitudes, as well as tailoring future interventions. In contrast, the equal gender representation in Class VIIIB provides a balanced subgroup for examining gender-specific responses to the awareness session.

Analysing gender dynamics within these classes allows researchers to better understand the varied impacts of health education, ensuring that future programs address the needs of all participants effectively.

**Age**

The participants’ age distribution was a critical variable in this study, aligning with the middle school demographic targeted by the intervention. Statistical analysis of the age data revealed:

**Mean Age:** 13.61 years, indicative of a typical middle-school cohort. **Median Age:** 14 years, reflecting the most common age among participants. **Mode Age:** 14 years, reinforcing the central tendency of the data.

**Age Range:** 12 to 15 years, highlighting the relatively narrow span of ages.

**Standard Deviation:** 0.95 years, illustrating limited variability within the group.

The consistency in age distribution underscores the homogeneity of the sample, which is advantageous for evaluating the intervention’s effectiveness. Such alignment between mean, median, and mode minimizes confounding variables, enhancing the reliability of the study’s conclusions.

**Socio-Demographic**

The educational qualifications of mothers showed significant disparities:

**No Formal Education:** 23 mothers (56%), indicating a significant gap in literacy.

**Primary Education:** 10 mothers (24%), reflecting basic education levels.

**Secondary Education:** 6 mothers (15%), indicating limited progression.

**Higher Secondary Education:** 1 mother (2%), showcasing minimal access to advanced education.

In terms of occupations, the majority around 27 mother’s (66%) were homemakers. Other roles included 5 daily wage workers (12%), 4 private sector employees (10%), 3 teachers (7%), and single instances of government employment and agricultural work (2% each).

**Religion and Caste**

Religion and caste are important socio-cultural factors that influence health awareness and behaviours. In the pilot study, participants were drawn from diverse religious and caste backgrounds. These variations provided an opportunity to examine how socio-cultural factors impact baseline knowledge and attitudes toward sickle cell disease, as well as the receptiveness to health education initiatives.

40

35

30

25

20

15

10

5

0

35

4

1

1

A B C F

**Religion** (A-Hinduism B- Islam C- Christianity F -Other)

**Total Participants**

**Total Participants**

| 20 | 1 | 19 | 8 | 13 |
| --- | --- | --- | --- | --- |
| 18 |  |  |  |  |
| 16 |  |  |  |  |
| 14 |  |  |  |  |
| 12 |  |  |  |  |
| 10 |  |  |  |  |
| 8 |  |  |  |  |
| 6 |  |  |  |  |
| 4 |  |  |  |  |
| 2 |  |  |  |  |
| 0 |  |  |  |  |
|  |  | | | |
|  | A B C D | | | |
|  | **Caste** | | | |
|  | (A- General Caste B- Other Backward Caste C- Scheduled Caste D - Scheduled Tribes) | | | |

**Tested For Sickle Cell Disease**

Testing for sickle cell disease was a significant focus of the pilot study. Participants were encouraged to consider the importance of early diagnosis in managing this genetic condition effectively. The study revealed notable shifts in participant’s willingness to undergo testing, demonstrating the impact of targeted health educational intervention.

The session addressed critical topics such as the purpose of sickle cell testing, its role in preventing complications, and the availability of accessible testing facilities. These discussions likely reduced fears and misconceptions, fostering a greater acceptance of the importance of testing.

Earlier 37 participants (90%) told that they didn’t tested for sickle cell disease. Additionally, the interactive format of the session allowed participants to ask questions and clarify doubts, further reinforcing their willingness to engage in proactive health measures.

This shift underscores the transformative potential of educational interventions in improving health behaviors and highlights the need for continued efforts to promote awareness and accessibility of sickle cell disease testing.

**Tested For Sickle Cell Disease**

4

37

**A** - No(37)

**B** - Yes (4)

**Effect of Health Awareness Session**

The health awareness session was a pivotal component of the study, focusing on improving participants’ understanding of sickle cell disease. The pre- and post-test results demonstrated substantial changes:

| **Willing to get tested** | **Yes** | **No** | **No Response** |
| --- | --- | --- | --- |
| **BEFORE Health education intervention** | 15 | 8 | 18 |
| **AFTER Health education intervention** | **34** | 5 | 2 |

**Before Health Educational Intervention:** Only 15 participants (37%) were willing to undergo testing for sickle cell disease, 8 participants (20%) were unwilling, and 18 (44%) provided no response.

**After Health Educational Intervention:** Willingness to test rose dramatically to 34 participants (83%), with only 5 participants (12%) remaining unwilling and 2 (5%) not responding.

This significant improvement highlights the effectiveness of the health awareness session in bridging knowledge gaps and addressing misconceptions. The session’s interactive and educational approach likely contributed to the marked shift in attitudes, underscoring the value of targeted health education initiatives.

# Preferences for Testing Locations

Participants’ preferences for sickle cell disease testing locations revealed practical considerations:

**Government Hospital:** 19 participants (46%), reflecting reliance on public health facilities.

**Schools:** 10 participants (24%), emphasizing the convenience of on-site testing.

**Nearest Primary Health Centre:** 3 participants (7%)

**Private Clinics:** 6 participants (15%), showing a minority preference for private care. Others: 1 participant (2%), representing unique preferences.

No Response: 2 participants (5%).

The preference for government hospitals suggests trust in affordable, reliable public health services. The high percentage favouring schools indicates that integrating health services

within an educational institution could improve accessibility and participation rates for improving health in holistic approach.

**Effectiveness of Health education intervention**

| **Paired Samples Statistics** | | | | | |
| --- | --- | --- | --- | --- | --- |
|  | | Mean | N | Std.  Deviation | Std. Error  Mean |
| Pair 1 | Pre Test  Knowledge | 5.3117 | 41 | 2.87163 | .44847 |
|  | Post Test  Knowledge | 12.5693 | 41 | 2.09062 | .32650 |
| Pair 2 | Pre Test Attitude | 1.44 | 41 | 9.214 | 1.439 |
|  | Post Test Attitude | 55.83 | 41 | 8.417 | 1.315 |


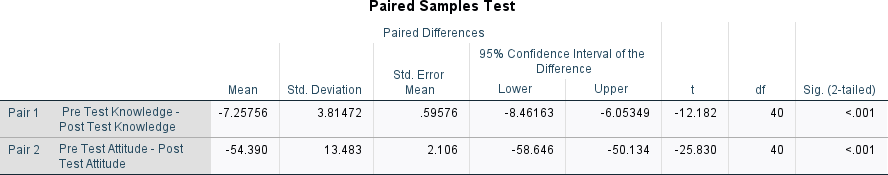


**p-value <0.001** -There is a significant difference in knowledge score before and after health educational intervention.

**p-value <0.001** - There is a significant difference in attitude score before and after health educational intervention.

The results from the paired samples t-test reveal significant improvements in both knowledge and attitude among the participants following the intervention. For knowledge, the average difference between pre-test and post-test scores is -7.26, indicating that post-test knowledge

scores were significantly higher. The 95% confidence interval for this difference ranges from

-8.46 to -6.05, excluding zero, which confirms the statistical significance of this improvement. Furthermore, the t-statistic of -12.182 and a p-value of less than 0.001 provide robust evidence that the observed increase in knowledge is not due to chance.

Similarly, for attitude, the average difference between pre-test and post-test scores is -54.39, showing a substantial increase in attitude scores after the intervention. The confidence interval for this difference, ranging from -58.65 to -50.13, also excludes zero, confirming the reliability of this improvement. The t-statistic of -25.83 and a p-value of less than 0.001 further indicate that this change in attitude is highly statistically significant.

The results of the analysis indicate a substantial and **statistically significant improvement in both knowledge and attitude scores following the health educational interventional session.** The p-values of less than 0.001 for both knowledge and attitude scores suggest that the changes observed were not due to chance, highlighting the effectiveness of the health educational intervention in enhancing participants’s understanding and shaping their attitudes. This outcome reflects the success of the health education intervention in effectively engaging participants and fostering positive shifts in both knowledge and behavioral perspectives, and strongly suggest that the intervention was effective in improving both knowledge and attitude, with the statistical evidence pointing to meaningful and consistent gains across both areas.

**Conclusion**

The pilot study offered critical insights into the effectiveness of health education interventions in improving knowledge and attitudes toward sickle cell disease. The health awareness session significantly increased participants’ willingness to undergo testing, underscoring the transformative potential of such initiatives. The findings also highlighted

preferences for accessible testing locations, such as government hospitals and schools, providing practical guidance for future program design.

**Reliability Statistics for Knowledge Questionnaire**

| **Reliability Statistics** | |
| --- | --- |
| **Cronbach's Alpha** | N of Items |
| **.722** | 21 |

The questionnaire used in this study is designed to assess the knowledge and attitude of adolescents in government school students regarding sickle cell disease (SCD). To ensure the reliability of the questionnaire, Cronbach’s alpha was calculated separately for the knowledge and attitude components. The knowledge questionnaire achieved a **Cronbach’s alpha of 0.722**, indicating acceptable internal consistency. This suggests that the knowledge-related items are sufficiently correlated to measure the underlying construct consistently.

**Reliability statistics for Attitude Questionnaire**

| **Reliability Statistics** | |
| --- | --- |
| **Cronbach's Alpha** | N of Items |
| **.990** | 15 |

On the other hand, the attitude questionnaire achieved a remarkably high **Cronbach’s alpha of 0.990**, indicating excellent internal consistency among the items assessing attitudes. This high value reflects the robustness of the attitude-related items in capturing the perspectives and opinions of adolescents towards SCD. Together, these reliability scores demonstrate that the questionnaire is a reliable tool for evaluating knowledge and attitudes among the target population, providing a solid foundation for the study's objectives.

# Details of expenses and source of funding

| **Category** | **Type** | **No. Required** | **Per Piece Cost** | **Total (in rupees)** |
| --- | --- | --- | --- | --- |
| Consumables | Questionnaire sets | 1500 | 10 | 15000 |
|  | IEC material | 3000 | 10 | 30000 |
| Screening  Kits | Hemoglobin Test  Kits | 400 | 50 | 20000 |
|  | Sickle Cell Test Kits | 400 | 50 | 20000 |
| Staff | Phlebotomist | 1 | 1000 per visit | 10000 |
| Transportation | visits | 30 | 3000 | 90,000 |
| Software | Jamovi | 1 | 0 | 0 |
|  | License for Atlas Ti  (1 year) | 1 | 7725 | 7725 |
| Expense | Miscellaneous |  |  | 10000 |
|  | Contingency Fund |  |  | 20000 |
| Total | | | | **2,22,725** |

Total Estimated Research-Related Expenses: **2,22,725 Rupees Source of Funding:**

I plan to apply to various funding agencies listed below to support the smooth execution of my project. If these applications are not successful, I am prepared to self-fund the project to ensure its continuation.

- 1. Government Grants:

Indian Council of Medical Research (ICMR): Proposal for funding to support research in sickle cell disease.

- 1. Non-Governmental Organizations (NGOs):

Local NGOs: Collaborate with local NGOs working on health and education in tribal and underserved communities.

- 1. Academic and Research Institutions
  2. Ministry of Tribal Affairs

# References

1. Thomson AM, McHugh TA, Oron AP, Teply C, Lonberg N, Vilchis Tella V, et al. Global, regional, and national prevalence and mortality burden of sickle cell disease, 2000–2021: a systematic analysis from the Global Burden of Disease Study 2021. Lancet Haematol. 2023 Aug;10(8):e585–99.
2. Elendu C, Amaechi DC, Alakwe-Ojimba CE, Elendu TC, Elendu RC, Ayabazu CP, et al. Understanding Sickle cell disease: Causes, symptoms, and treatment options. Medicine. 2023 Sep 22;102(38):e35237.
3. Kato GJ, Piel FB, Reid CD, Gaston MH, Ohene-Frempong K, Krishnamurti L, et al. Sickle cell disease. Nat Rev Dis Primers. 2018 Mar 15;4(1):18010.
4. Kato GJ, Gladwin MT, Steinberg MH. Deconstructing sickle cell disease: Reappraisal of the role of hemolysis in the development of clinical subphenotypes. Blood Rev. 2007 Jan;21(1):37–47.
5. Platt OS, Brambilla DJ, Rosse WF, Milner PF, Castro O, Steinberg MH, et al. Mortality In Sickle Cell Disease -- Life Expectancy and Risk Factors for Early Death. New England Journal of Medicine. 1994 Jun 9;330(23):1639–44.
6. Government of Jharkhand. https://[www.jharkhand.gov.in/home/AboutTribals](http://www.jharkhand.gov.in/home/AboutTribals) . 2024.
7. Jain D, Gupta M, Madkaikar M, Jena RK, Khargekar N, Saraf SL, et al. Sickle cell disease in India: current status and progress. Vol. 11, The Lancet Haematology. Elsevier Ltd; 2024. p. e322–3.
8. Colah RB, Mukherjee MB, Martin S, Ghosh K. Sickle cell disease in tribal populations in India. Indian J Med Res. 2015 May;141(5):509–15.
9. Dr. Sona Pathak, Dr. Mushtaque Ahmad Ansari, Dr. Suraj Sinha. PREVALENCE OF SICKLE CELL DISORDER IN JHARKHAND: A RETROSPECTIVE CASE STUDY. Glob J Res Anal. 2020 Jun;9(6).
10. Albagshi M, Altaweel H, AlAlwan M, AlHashem H, Albagshi M, Habeeb F, et al. Sickle cell disease awareness among school children in Saudi Arabia. International Journal of Medicine in Developing Countries. 2019;998–1001.
11. Bindhani BK, Nayak JK. Comparative assessment of quality of life among adolescents with sickle cell disease and sickle cell trait: evidence from Odisha, India. J Community Genet. 2024 Jun;15(3):311–8.
12. Namukasa S, Maina R, Nakaziba S, Among G, Asasira L, Mayambala P, et al. Prevalence of sickle cell trait and needs assessment for uptake of sickle cell screening among secondary school students in Kampala City, Uganda. PLoS One. 2024 Jan 19;19(1):e0296119.
13. Arishi WA, Alhadrami HA, Zourob M. Techniques for the Detection of Sickle Cell Disease: A Review. Micromachines (Basel). 2021 May 5;12(5).
14. Bindhani BK, Devi NK, Nayak JK. Knowledge, awareness, and attitude of premarital screening with special focus on sickle cell disease: a study from Odisha. J Community Genet. 2020 Oct 18;11(4):445–9.
15. Surti SB, Parmar A, Babu B V., Patel GM, Godara N, Mishra U, et al. Endline assessment of knowledge about sickle cell disease among the tribal community of Chhotaudepur district of Gujarat. J Community Genet. 2024 Feb 9;15(2):187–94.
16. Tripathy P, Nair N, Mahapatra R, Rath S, Gope RK, Rath S, et al. Community mobilisation with women’s groups facilitated by Accredited Social Health Activists (ASHAs) to improve maternal and newborn health in underserved areas of Jharkhand and Orissa: study protocol for a cluster-randomised controlled trial. Trials. 2011 Dec 25;12(1):182.
17. World Health Organization. https://[www.who.int/southeastasia/health-topics/adolescent-](http://www.who.int/southeastasia/health-topics/adolescent-) health#:~:text=WHO%20defines%20’Adolescents’%20as%20individuals,15%2D24%20year%2 0age%20group. 2024.
18. Smith PG MRRD editors. Types of intervention and their development. In: Field Trials of Health Interventions: A Toolbox 3rd edition. 3rd ed. 2015.
19. Guidelines For Multipurpose Health Worker (Male) 2010 Government of India Ministry of Health and Family Welfare Nirman Bhawan, New Delhi [Internet]. [cited 2024 Aug 13]. Available from: https://nhm.gov.in/images/pdf/guidelines/nrhm-guidelines/guidlines_mphw- m.pdf
20. United Nations. https://sdgs.un.org/goals. 2024.

# Course work detail template

| **Serial No.** | **Course name** | **Credits** | **Status** |
| --- | --- | --- | --- |
| 1. | Research Methodology | 4 | Completed |
| 2. | Research and Publication Ethics | 2 | Registered |
| 3 | International Workshop on Qualitative Data  Analysis | 1 | Completed |
| 4. | Qualitative Research Methods and Research Writing | 3 | Ongoing |
| 5. | Biostatistics in Public Health Specialization | 4 | Registered |
| 6. | Scientific Writing in Health Research | 1 | Completed |

**Registered for**

**Workshop on Systematic Review and Meta-Analysis ,** from 22^nd^ – 29^th^ March ,2025 by Merit India

1. **Similarity check report**


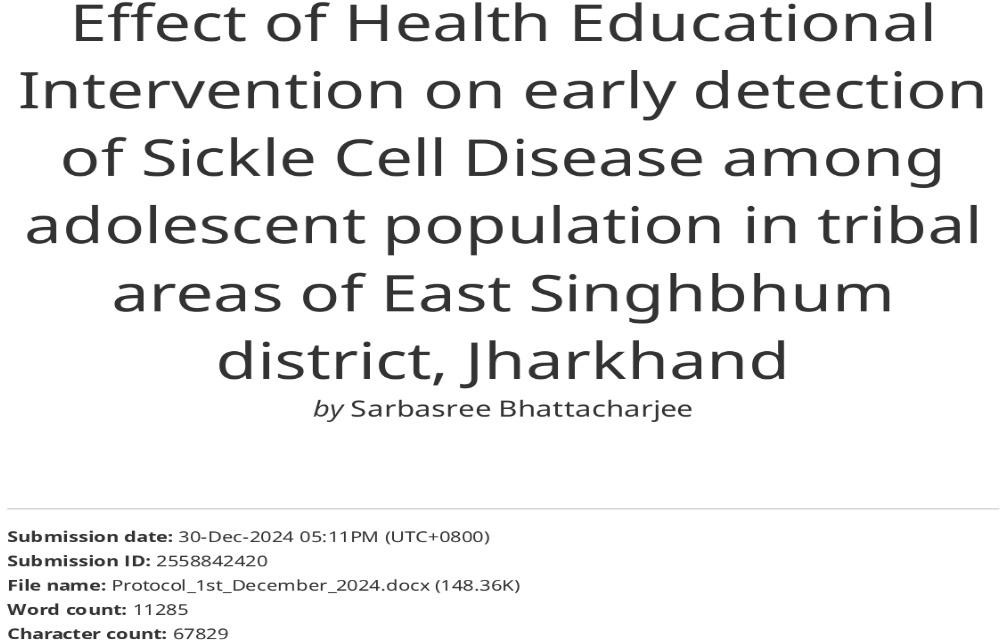

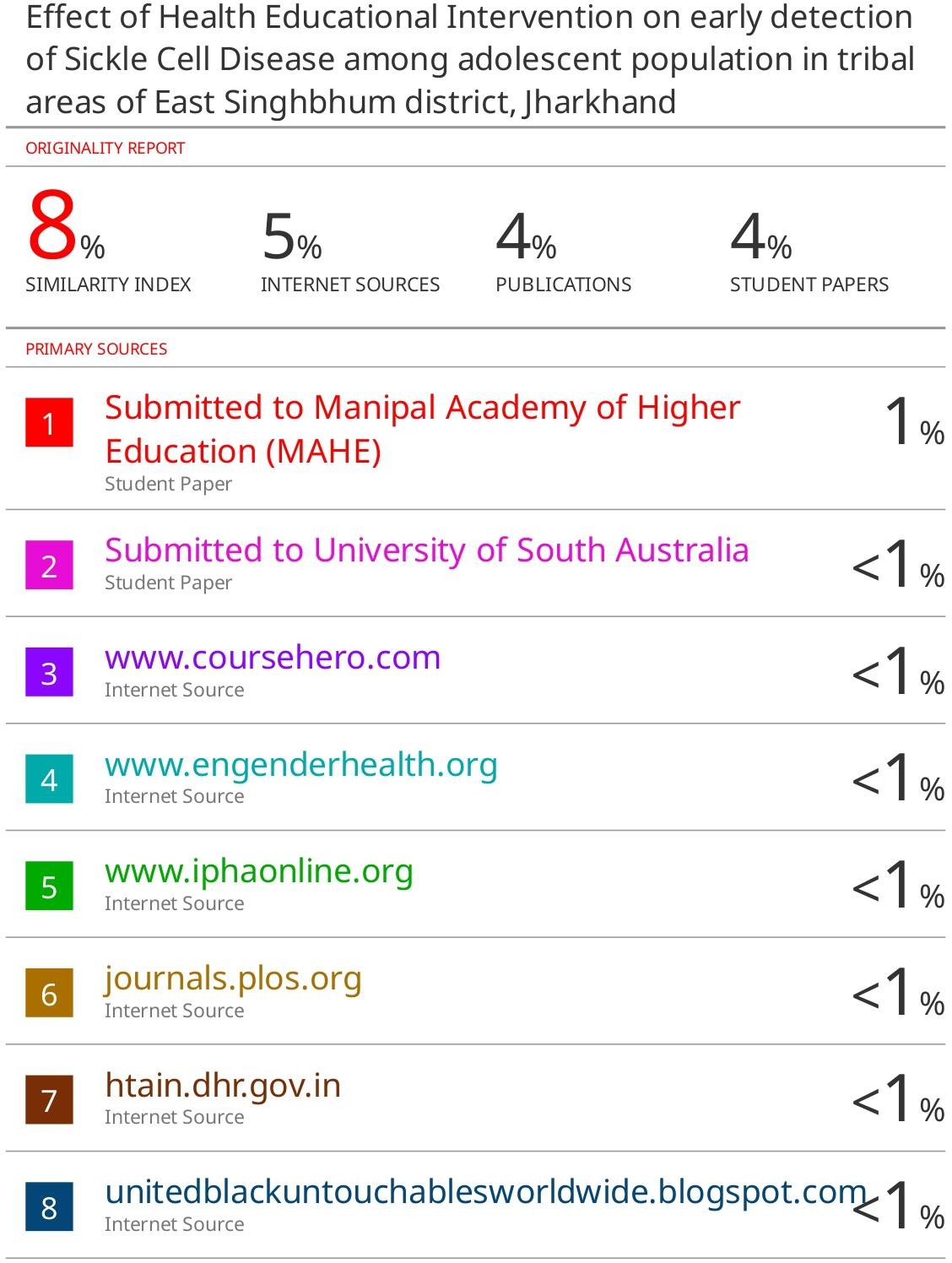

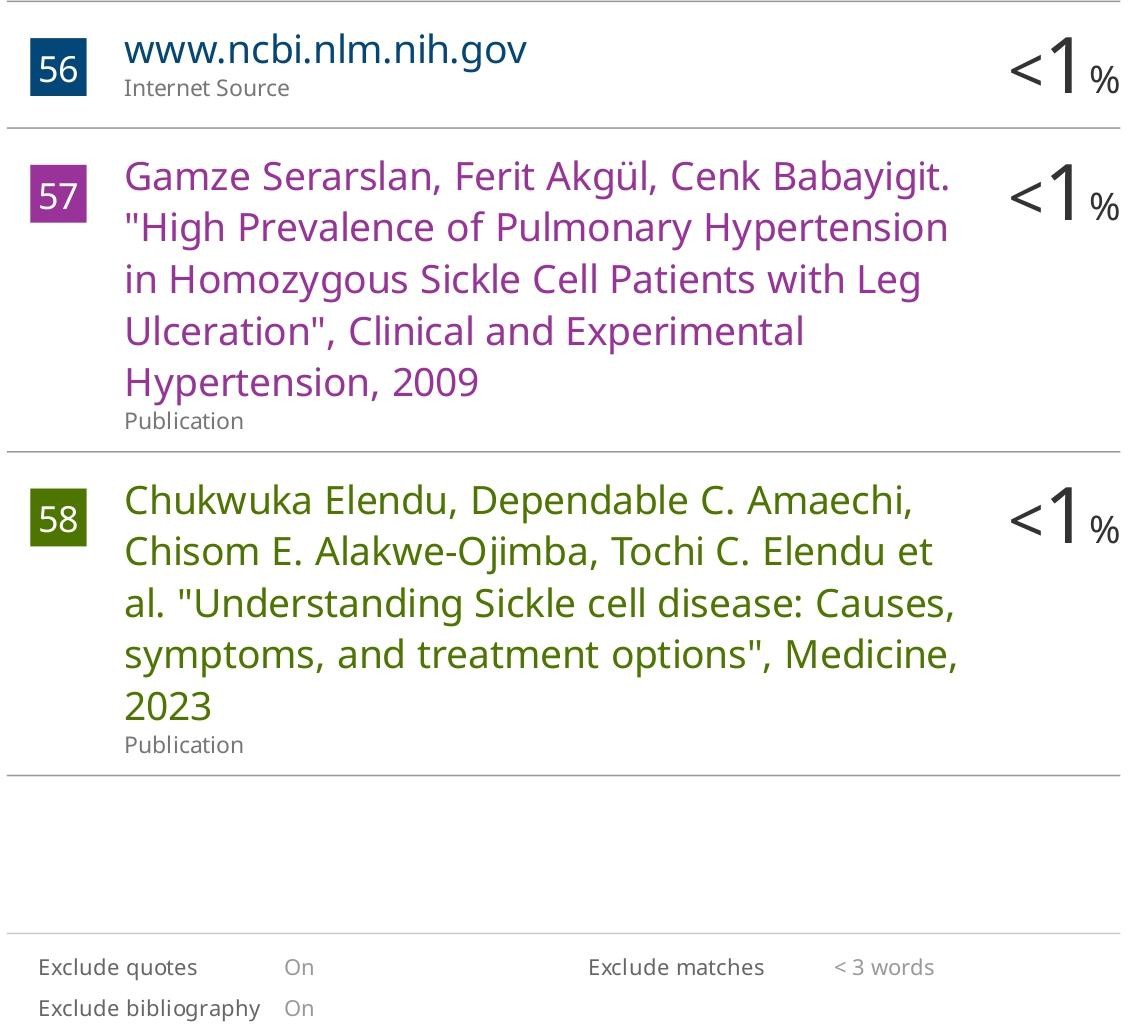

Supplement: S1 File — (DOCX) [file pone.0345849.s001.docx]
